# Tumor microenvironment remodeling by STING agonism sensitizes endothelial cells to cytotoxic anti-PD-L1/L2 antibody

**DOI:** 10.1186/s13046-026-03711-9

**Published:** 2026-04-14

**Authors:** Ahmad Salameh, Elisabetta Bolli, Manuela Iezzi, Christine Gagliardi, Laura Conti, Chiara Cossu, Paul Blezinger, Alessia Lamolinara, Andrew Lewis, Michael A. Curran, Federica Cavallo, Federica Pericle

**Affiliations:** 1 ImmunoGenesis, Inc., Houston, TX USA; 2 Department of Molecular Biotechnology and Health Sciences, Laboratory of OncoImmunology, Molecular Biotechnology Center “Guido Tarone”, University of Turin, Piazza Nizza 44b, Turin, 10126 Italy; 3 Department of Neurosciences, Imaging and Clinical Sciences, Laboratory of Experimental Pathology and Precision Medicine, Center for Advanced Studies and Technology (CAST), “G. d’Annunzio” University of Chieti-Pescara, Chieti, Italy; 4 Department of Pathology, Eusoma Breast Centre, “G. Bernabeo” Hospital Ortona, ASL2 Abruzzo, Ortona, Italy; 5 Department of Immunology, The University of Texas MD Anderson Cancer Center (MADCC), Houston, TX USA

**Keywords:** STING agonist, PD-L1/PD-L2 dual blockade, Tumor microenvironment, ADCC, ADCP, Vascular remodeling, Immune-excluded tumors, Cancer immunotherapy

## Abstract

**Background:**

Checkpoint inhibitors targeting PD-1 and PD-L1 have revolutionized cancer immunotherapy; however, their efficacy remains limited in immune-excluded tumors characterized by scarce T-cell infiltration and a profoundly immunosuppressive tumor microenvironment (TME). Activation of the stimulator of interferon genes (STING) pathway represents a promising strategy to overcome immune exclusion by promoting TME remodeling and immune cell recruitment. This study investigated the therapeutic potential of combining 8803, a potent STING agonist with broad cross-species activity, and 27907, a novel Fc-engineered dual-specific antibody targeting PD-L1 and PD-L2, designed to enhance antibody-dependent cytotoxicity and phagocytosis.

**Methods:**

The activity of 8803 was assessed in human and murine STING reporter cell lines and in co-culture systems with tumor, endothelial, and immune cells. The Fc-mediated effector functions of 27907 were characterized through ADCC and ADCP reporter bioassays. Antitumor efficacy was evaluated in B16-PD-L2 melanoma (C57BL/6) and TS/A mammary carcinoma (BALB/c) mouse models treated with intratumoral 8803 and/or systemic 27907. Tumor growth and survival were monitored, and immune and vascular remodeling were analyzed by flow cytometry, immunohistochemistry, and immunofluorescence. Statistical analyses were performed using two-way ANOVA with Bonferroni post-test for tumor growth, Mantel–Cox log-rank test for survival, and unpaired Student’s t-test or one-way ANOVA for in vitro and ex vivo data.

**Results:**

The combination of 8803 and 27907 resulted in significant tumor growth inhibition and prolonged survival compared with single-agent treatments. STING activation by 8803 remodeled the TME by reducing intratumoral M2-like macrophages and mature regulatory dendritic cells (mregDCs) while enhancing T-cell and myeloid cell infiltration. It also induced PD-L1 and PD-L2 upregulation on tumor and endothelial cells. The dual-specific antibody 27907 efficiently mediated antibody-dependent cellular cytotoxicity and phagocytosis, leading to selective endothelial cell killing, vascular disruption, extensive necrosis, and enhanced immune infiltration in the combination treatment group.

**Conclusions:**

Dual PD-L1/PD-L2 blockade synergizes with STING pathway activation to promote immune and vascular remodeling, resulting in superior antitumor efficacy in preclinical tumor models. These findings provide a strong rationale for the clinical development of combination strategies that integrate STING agonists with cytotoxic checkpoint antibodies to overcome immune exclusion and enhance cancer immunotherapy outcomes.

**Supplementary Information:**

The online version contains supplementary material available at 10.1186/s13046-026-03711-9.

## Background

Activation of the stimulator of interferon genes (STING) pathway by cyclic dinucleotides (CDNs) induces potent production of pro-inflammatory cytokines and chemokines, particularly type I interferons (IFNs) via transcription factors such as nuclear factor-κB (NF-κB), interferon regulatory factor (IRF) activation (IRF3/IRF7), and signal transducer and activator of transcription (STAT) 6 [1]. This immune activation enhances CD8⁺ T-cell priming and promotes their infiltration into tumors, thereby facilitating adaptive antitumor immunity [2, 3]. Furthermore, STING activation reprograms tumor-associated macrophages (TAMs) toward an inflammatory, tumor-suppressive phenotype, supporting antitumor activity through macrophage-mediated phagocytosis, natural killer (NK)-cell recruitment, and the reversal of therapy resistance [4–7]. Despite these promising immunomodulatory properties, clinical STING agonists have demonstrated limited efficacy as monotherapies, particularly in tumors characterized by a dense, immunosuppressive microenvironment [2, 8, 9].

One potential reason is the persistence of inhibitory signals mediated by PD-L1 and PD-L2, the ligands for programmed cell death protein-1 (PD-1), which are expressed not only on tumor cells but also on various stromal and immune cells within the tumor microenvironment (TME), including TAMs, mature dendritic cells enriched in immunoregulatory molecules (mregDCs), regulatory T cells (Tregs), myeloid-derived suppressor cells (MDSCs), cancer-associated fibroblasts, and endothelial cells [10–15].

These ligands engage PD-1 on T cells to suppress immune activation, forming a major barrier to effective anti-tumor immunity even in the context of STING-induced inflammation. Conversely, many tumors resistant to immune checkpoint blockade (ICB) [16], particularly those considered immunologically “cold,” exhibit limited immune infiltration and poor antigen presentation, deficiencies that STING activation could potentially overcome [8, 17–19].

Mechanisms underlying resistance to both STING agonists and ICB not only underscore their individual limitations but also provide a compelling rationale for their combined use to achieve synergistic anti-tumor effects. These insights have generated considerable interest in combining STING agonists with ICB, given their potentially complementary mechanisms of action [3, 9, 17, 20].

However, the disappointing outcomes of clinical trials conducted to date [20] suggest that currently available agents may lack the potency or functional attributes necessary to effectively remodel the immunosuppressive TME. This highlights a critical need for next-generation molecules with improved pharmacologic properties and mechanisms of action capable of fully realizing the therapeutic promise of this combination strategy.

To address this gap, we evaluated a novel therapeutic strategy combining two recently developed agents with synergistic and complementary properties. The first, 8803, is a potent CDN STING agonist that has previously demonstrated superior activity compared to the clinical benchmark ADU-S100 [9, 21, 22]. The second agent, 27907, is a dual-specific antibody targeting both PD-L1 and PD-L2, engineered with Fc modifications (GASDIE) [23] to enhance antibody-dependent cellular cytotoxicity (ADCC) and antibody-dependent cellular phagocytosis (ADCP) [24, 25]. This design enables not only effective blockade of PD-1 ligands but also active elimination of immunosuppressive cells within the TME. We hypothesized that the combination of these agents would complement one another to potentiate remodeling of the TME, enhance immune cell infiltration, and drive robust anti-tumor immunity.

In murine models of melanoma (B16-PD-L2) [24–26] and mammary carcinoma (TS/A) [27, 28], 8803 alone induced important TME remodeling by shifting TAMs toward an inflammatory phenotype and promoting immune infiltration. However, only the addition of 27907 enabled selective depletion of PD-L1⁺/PD-L2⁺ endothelial and immunosuppressive myeloid cells, resulting in vascular disruption, widespread tumor necrosis, and a significant survival benefit. These findings demonstrate that cytotoxic targeting of PD-L1/PD-L2 synergizes with potent STING activation to overcome multiple layers of immune resistance within the TME.

## Materials and methods

### Cell lines

Mouse brain endothelial cells bEnd.3 (BEND3; Cat#CRL-2299) and B16F10 (Cat#CRL-6475) cell lines were purchased from ATCC, expanded, aliquoted, and passaged fewer than 10 times after resuscitation. Cells were cultured in RPMI-1640 medium (ThermoFisher Scientific) supplemented with 10% fetal bovine serum (FBS; Gibco) and 1% penicillin-streptomycin and 2mM L-glutamine (ThermoFisher Scientific). TS/A cells, established from a HER2-positive spontaneous mammary tumor arising in a BALB/c female retired breeder [27], were cultured in RPMI-1640 with 10% FBS and 1% penicillin-streptomycin [29]. B16-PD-L2 cells were generated by transfecting B16F10 cells with mouse PD-L2, followed by fluorescence-activated cell sorting [24–26]. These cells were maintained in RPMI-1640 supplemented with 10% FBS, MEM Non-Essential Amino Acids 1X (Gibco, Cat#11140050), 1 mM sodium pyruvate (Gibco, Cat#11360070) and 1% penicillin-streptomycin. The cell lines THP1-Dual™ (Cat#thpd-nfis), THP1-Dual™ STING knockout (THP1-KO; Cat#thpd-kostg), 293-Dual™ (Cat#293d-mstg), THP1/CXCL10 (Cat#thpb-ip10kilc), 293/IFN-α/β Reporter (Cat#hkb-ifnabv2) and Raji-PDL1 (Cat#Raji-hpdl1) were purchased from InvivoGen and maintained according to the manufacturer’s instructions. U2940 (kindly provided by Curran Laboratory, MDACC) and human umbilical vein endothelial cells (HUVEC; ATCC, PCS-100-010™) were maintained in Endothelial Cell Growth Medium (EGM-2; ATCC, Cat#CC-3162) supplemented with the EGM™-2 BulletKit™ components, following the manufacturer’s instructions. All cell lines were cultured at 37 °C in a humidified atmosphere containing 5% CO₂ and tested negative for mycoplasma contamination using the MycoAlert™ Mycoplasma Detection Kit (Lonza, Cat#LT07-318). bEnd.3 and HUVEC cells stably expressing GFP-Luciferase were generated using lentiviral transduction.

### 8803 and other STING agonists

Compound 8803 is a synthetic STING agonist with a chemical structure and mechanism of action optimized for potent activation of both human and mouse STING variants *in vitr*o [21]. ADU-S100 (MIW815), a synthetic CDN STING agonist currently in clinical development, was purchased from InvivoGen (Cat#tlrl-nacda2r-01) and activates the STING pathway by mimicking endogenous CDNs. 2′3′-cGAMP, the endogenous mammalian cyclic GMP-AMP produced by cGAS in response to cytosolic DNA, was obtained from InvivoGen (Cat#tlrl-nacga23-02) and served as a natural ligand and potent STING activator. 2′3′-cGMP linear (InvivoGen; Cat#tlrl-nagpap), a linear analog of cyclic GMP-AMP with modified backbone chemistry, was used as negative control to assess STING activation dynamics and specificity. Mouse and human recombinant IFN-α (Acrobiosystems; Cat#IFA-M52H3, Cat#IFA-H5258), IFN-β (MedChemExpress; Cat#HY-P73130, Cat#HY-P73129), and IFN-γ (Acrobiosystems; Cat#IFG-M82E3, Cat#IFG-H4211). All compounds were used according to the manufacturers’ instructions.

### Testing 8803 potency on STING reporter cell lines

To evaluate the potency of the STING agonist 8803, THP1-Dual™ (expressing human STING) and 293-Dual™ (expressing mouse STING) reporter cell lines were used. Cells were seeded at 2 × 10⁵ cells/well in 96-well plates, followed by treatment with 8803 and other STING-agonist compounds at the indicated concentrations, and cultured for 18 h. Supernatants were then collected and assessed for activation of IRF or NF-κB pathways. IRF-dependent luciferase activity was measured using QUANTI-Luc reagent (Cat#rep-qlc4lg1) and NF-κB–driven SEAP activity was quantified using QUANTI-Blue Solution (Cat#rep-qbs) both according to the manufacturer’s protocols (InvivoGen). Luminescence and absorbance (optical density; O.D.) were measured using the BioTek Cytation™ 5 Cell Imaging Multi-Mode Read.

### Anti–PD-L1/PD-L2 monoclonal antibody 27907

The monoclonal antibody 27907 is a dual-specific antibody engineered to simultaneously target the immune checkpoint ligands PD-L1 and PD-L2. It contains a human IgG1 constant region incorporating the GASDIE mutations [23], which enhance ADCC and ADCP, thereby potentiating effector functions against PD-L1/PD-L2–expressing cells [24–26]. As a negative control for ADCC and ADCP, an isotype variant of 27907 carrying LALA-PG mutations (L234A/L235A/P329G) in the Fc region, which abrogate Fc-mediated effector functions [30], was also generated. In addition, a murine version of 27907 was generated in the mouse IgG2a backbone for use in mouse studies.

### PD-1/PD-L1 and PD-1/PD-L2 blockade bioassay

The PD-1/PD-L1 and PD-1/PD-L2 blockade bioassay kits were purchased from Promega (Cat# J1250 and CS187135-1, respectively) and used according to the manufacturer’s instructions. The kits contain Jurkat T cells stably expressing human PD-1 and NFAT-induced luciferase, Chinese hamster ovary (CHO)-K1 cells stably expressing human PD-L1 (CHO/PD-L1) or PD-L2 (CHO/PD-L2), and a cell surface protein designed to activate Jurkat cells in an antigen-independent manner. When the two cell types are co-cultured, the PD-1/PD-L1 or PD-1/PD-L2 interaction inhibits TCR signaling and NFAT-mediated luciferase activity. Addition of an antibody that blocks either PD-1, PD-L1, or PD-L2 releases the inhibitory signal and results in TCR signaling and NFAT-mediated luciferase activity. The clinically approved antibody pembrolizumab (anti–PD-1) and an isotype-matched antibody were used as positive and negative control, respectively.

### Bio-layer interferometry binding kinetics assay

Binding kinetics were measured using bio-layer interferometry on an Octet instrument. Association and dissociation between the antibody and immobilized proteins were monitored in real time. The resulting sensorgrams were analyzed using Octet analysis software to determine the association rate constant (k_on), dissociation rate constant (k_off), and equilibrium dissociation constant (Kd).

### ADCC/ADCP bioassays

ADCC and ADCP were assessed using commercially available reporter bioassay kits from Promega: human FcγRIIIa ADCC Reporter Bioassay (Cat#G7015), and human FcγRIIa ADCP reporter bioassay (Cat#GA1341). All assays were performed according to the manufacturer’s instructions. Effectors consisted of Jurkat reporter cells engineered to express human FcγRIIIa, or FcγRIIa-H, enabling luciferase-based quantification of Fc receptor engagement. As target cells, CHO/PD-L1, CHO/PD-L2, U2940, Raji/PD-L1, B16-PD-L2 and HUVEC cells were used. In these assays, effector activity was evaluated in the presence of test antibody 27907. Rituximab (anti-CD20) was used as a positive control to confirm assay performance, while a matched non-binding isotype control antibody served as a negative control.

### Flow cytometry (FACS) analysis of PD-L1 and PD-L2 expression and cell viability in bEnd.3 and HUVEC endothelial cells

bEnd.3 and HUVECs were seeded into 6-well plates at approximately 85% confluency and incubated overnight at 37 °C in a humidified atmosphere containing 5% CO₂. The following day, cells were treated with 8803 (at concentrations of 3, 10, and 30 µg/mL), IFN-α (10 µg/mL), IFN-β (10 µg/mL), and IFN-γ (50 µg/mL) for 18 h. After treatment, cells were detached using 0.05% trypsin-EDTA, washed with PBS containing 2% FBS, and resuspended in FACS buffer (PBS with 2% FBS). To assess surface PD-L1 and PD-L2 expression, cells were incubated with anti-mouse PD-L1 (10 F.9G2.1, BioLegend) and PD-L2 (TY25, BioLegend) antibodies and anti-human PD-L1 (29E.2A3, BioLegend) and PD-L2 (MIH18, BioLegend) antibodies, or the corresponding isotype control for 30 min at 4 °C in the dark. Following antibody staining, cells were washed and resuspended in FACS buffer containing 7-Aminoactinomycin D (7-AAD; BioLegend, 0.5 µg/mL) to evaluate cell viability. Samples were acquired using a Sony SA3800 flow cytometer, and data were analyzed using the manufacturer’s software. Cytokine responses were assessed using luciferase-based reporter cell lines engineered to respond to specific cytokines. To evaluate activation of the interferon pathways, specifically induction of IFN-α/β and CXCL10 production, reporter cell lines obtained from InvivoGen were seeded in 96-well plates and stimulated with supernatants collected from 8803-stimulated bEnd.3 and HUVEC cells. Following incubation (18–20 h), cytokine induction was quantified using a luminometric assay, according to the manufacturer’s instructions. Background signal from untreated controls was subtracted, and fold induction was calculated relative to baseline. All assays were performed in technical replicates, and data were normalized to internal standards or appropriate positive controls.

### Generation of monocytic-enriched myeloid cells from mouse splenocytes

Spleens were collected from C57BL/6 mice and mechanically dissociated through a 70-µm cell strainer into 5 mL of PBS supplemented with 2% heat-inactivated FBS. The resulting single-cell suspensions were centrifuged at 400 × g for 5 min at 4 °C. Red blood cells were lysed by resuspending the pellet in pre-warmed tris-ammonium chloride for 30 s at 37 °C, followed by immediate washing with PBS. After a second filtration through a fresh 70-µm strainer, cells were resuspended at a concentration of 5 × 10⁶ cells/mL in RPMI-1640 medium supplemented with 10% heat-inactivated FBS and recombinant mouse GM-CSF (PeproTech, at 20 ng/mL), IL-2 (MedChemExpress, 20 ng/mL), and IL-6 (MedChemExpress, 20 ng/mL) unless otherwise specified. Cultures were maintained at 37 °C and fresh medium containing GM-CSF was added on day 3. Monocytic-enriched myeloid cells were harvested between days 3 and 5 and subsequently used in co-culture assays to assess the cytotoxic effects of 8803 on endothelial or cancer cells.

### PBMCs preparation

PBMCs were isolated from heparinized peripheral blood obtained from Gulf Coast Regional Blood Center (Houston, TX) using the SepMate™ isolation system (STEMCELL Technologies) according to the manufacturer’s instructions. Isolated PBMCs were collected, counted, and cryopreserved at a density of 2.5 × 10^7^ cells/mL in freezing medium (CryoStor^®^ CS10#07930) until use in co-culture experiments.

### Testing of 8803, 27907, and their combination in co-culture systems of bEnd.3 and HUVECs

bEnd.3 and HUVEC cells stably expressing GFP-Luciferase were cultured in T75 flasks (Greiner Bio-One) using their respective complete growth media until reaching 85–90% confluency. For co-culture experiments, cells were harvested, counted, and seeded into 96-well plates at a density of 1 × 10^4^ cells per well. After allowing cells to adhere and grow for 18 h, they were pre-treated according to experimental conditions. Following pre-treatment, monocytic-enriched myeloid cells were added to bEnd.3 cultures, while human PBMCs pre-treated with recombinant human GM-CSF, IL-2 and IL-6 were added to HUVEC cultures. Co-cultures were established at 5:1 effector-to-target (E: T) cell ratio in presence of 8803, 27907 or combination of both. At the end of the co-culture period, cell viability was assessed by measuring luciferase activity in the target cells. Luminescence was quantified as relative luminescence units (RLU) using a microplate reader.

### Western blotting

Cells were incubated for 1 h with 10 µg/mL of 8803 and the commercial STING agonists 2′3′cGAMP and ADU-S100. Cells were then lysed for protein extraction using a Triton lysis buffer (50 mM Tris-HCl pH 7.6, 120 mM NaCl, 1% Triton X-100) supplemented with 1 mM PMSF, 1 mM NaVO_4_, 1 mM NaF, and a protease inhibitors cocktail (Sigma-Aldrich; Cat#P8340) for 20 min on ice. Samples were scraped and collected, then centrifuged for 10 min at 14,000 g. Supernatants containing proteins were harvested and quantified using the Pierce™ BCA Protein Assay Kit (Thermo-Fisher Scientific; Cat#23225). Proteins were diluted with 4X Laemmli loading buffer (BioRad; Cat#1610747) plus β-mercaptoethanol (1:10) to reach a concentration of 1 µg/µL and then boiled at 95 °C for 10 min. Subsequently, 40 µg of proteins were loaded onto a 4–20% polyacrylamide Precast Gel PROTEAN TGX™ (Bio-Rad; Cat#456–1094). After separation through the gel, proteins were transferred to a PVDF membrane using the Trans-Blot Turbo RTA mini 0.45 μm PVDF transfer kit (Bio-Rad; Cat#17904274) for 30 min. Non-specific binding sites were blocked using 5% non-fat milk (Santa Cruz Biotechnology.; Cat#sc-2325) in 1X Tris Buffered Saline supplemented with 0.1% Tween 20 (T-TBS) for 1 h at room temperature. The membranes were incubated overnight at 4 °C with mouse anti-vinculin (produced in-house, 1:8000), rabbit anti-STING (Cat#13647, Cell Signaling Technology, 1:1000), rabbit anti-p-TBK1/TBK1 (Cat#5483, Cat#3504, Cell Signaling Technology, 1:5000), or rabbit anti-p-IRF3/IRF3 (Cat#29047, Cat#4302, Cell Signaling Technology, 1:5000) antibodies. This was followed by a 1-hour incubation with HRP-linked goat anti-mouse (Cat#A0545, Sigma-Aldrich, 1:2000) or with HRP-linked goat anti-rabbit (Cat#A4416, Sigma-Aldrich, 1:2000 or 1:300000) antibodies. Signals were detected by reaction with HRP substrate (Westar ECL substrate Antares, Cat#XLS0142, or Hypernova, Cat#XLS149, Cyanagen) and images were acquired using a Chemidoc Touch Imaging System (Bio-Rad).

### In vivo studies

Seven-week-old female BALB/c (BALB/cAnNCrl) and C57BL/6 (C57BL/6NCrl) mice were obtained from Charles River Laboratories. All animal procedures were conducted in accordance with the Institutional Animal Care and Use Committee (IACUC). C57BL/6 mice were injected subcutaneously (s.c.) with 1 × 10^5^ B16-PD-L2 cells in left flank, while BALB/c mice were injected orthotopically with 1 × 10^5^ TS/A cells into the fourth left mammary gland. Mice were then inspected daily for tumor appearance. Tumors were measured twice a week with calipers in two perpendicular diameters. The following formula was used to calculate tumor volume [31]: length (mm) x width (mm) x width (mm) x 0.5236 = volume (mm^3^). When the tumor reached 75–100 mm^3^, mice were randomized in the treatment groups. 27907 (200 µg per dose intraperitoneal; i.p.) or PBS were administered on days 11, 14, 17, and 21 after tumor cell challenge, while the STING agonist 8803 (10 µg per dose intratumoral; i.t.) was delivered on days 11 and 14. Tumors, spleens, and tumor draining lymph nodes (TDLNs) were harvested on day 18 from 3 to 4 mice per group and processed for TME analysis using histology, immunohistochemistry (IHC), immunofluorescence (IF), and FACS analysis. All other mice were euthanized when the tumor reached 500-1,500 mm^3^. In a separate experiment, C57BL/6 mice (*n* = 6–7 mice/group) were injected with B16-PD-L2 cells and when tumor reached an average volume of 110 mm^3^, were treated i.p. with PBS, 27907 or the Fc-silenced variant 27907-LALA-PG (400 µg/dose on days 11, 14, 17, and 21 after tumor challenge). The STING agonist 8803 (10 µg per dose, i.t.) was delivered on days 11 and 14. Experimenters were not blind to group assignment and outcome assessment.

### FACS analysis

B16-PD-L2 or TS/A tumors were harvested, finely minced with sterile blades, and digested at 37 °C for 30 min on an orbital shaker in RPMI-1640 medium containing collagenase I and IV (1 mg/mL each; Merck). Following digestion, cell suspensions were washed in RPMI-1640 supplemented with 10% FBS, passed through a 70-µm cell strainer, then centrifuged at 400 × g for 10 min. Spleens and TDLNs were harvested and smashed onto a 70-µm cell strainer, followed by centrifugation at 400 × g for 10 min. All the resulting cell pellets were incubated in erythrocyte lysis buffer (155 mM NH₄Cl, 15.8 mM Na₂CO₃, 1 mM EDTA, pH 7.3) for 5 min at room temperature to remove red blood cells. Cells were then washed again in RPMI-1640 with 10% FBS, and 1 × 10⁶ cells were resuspended in 2% FBS in PBS. Fc receptors were blocked using anti-mouse CD16/CD32 antibody (ThermoFisher Scientific). Surface staining was performed at 4 °C for 30 min using various combinations of the following fluorophore-conjugated antibodies: CD45-VioGreen, CD11b-FITC, CD11c-APC, F4/80-PE-Vio770, Ly6C-APC/Vio770, Ly6G-VioBlue, CD3-FITC, CD4-APC/Vio770, CD8-VioBlue, PD-1-APC (from Miltenyi Biotec) and CD44-PE, CD206-PE, PD-L1-PE, PD-L1-APC, PD-L1-PeFire700, PD-L2-PE, PD-L2 BUV563 or PD-L2-PECF594 and CD86 (GL1, Brilliant Violet, BV, 711) from BioLegend. CD206 (BV 650), PD-L1 (CD274, PE-Fire 700), CD14 (BV 421), CD8a (BV 510), FoxP3 (PE) were from BioLegend; CD45 (AlexaFluor 647), F4/80 (Brilliant Ultraviolet, BUV, 496), PD-L2 (CD273, BUV 563), CD11c (BV786), CD86 (BV 711), CD3 (BUV 395), PD-1 (RealBlue 705), were from BD Biosciences; Fixable Live/Dead (Live/Dead Fix Near IR 775), Ly-6G (BUV 737), CD45 (BV 786), CD4 (BUV 737), and CD44 (BUV 615) (from Thermo Fisher); CD11b (StarBright UV445) and CD19 (StarBright Blue 615) were from Bio-Rad; Ly-6 C (Vio Bright 515) and CD25 (Vio Bright 667) were from Miltenyi Biotec. Representative gating strategies are shown in Supplementary Fig. 4B. Briefly, live singlet CD45⁺ cells were first selected and then subdivided using lineage markers. Monocytic (m)MDSCs were defined as CD11b⁺Ly6C⁺Ly6G⁻, granulocytic (g)MDSCs as CD11b⁺Ly6G⁺Ly6C^low^, and TAMs as CD11b⁺Ly6G⁻Ly6C⁻F4/80⁺. Quantification of PD-L1, PD-L2, and CD206 expression was performed calculating (i) the percentage of positive cells above the fluorescence-minus-one (FMO) or isotype control threshold in the corresponding parent populations, and (ii) the Mean Fluorescence Intensity (MFI) for the positive populations, to assess expression level per cell. Data were acquired using a Sony 3800 or a BD FACSVerse flow cytometers and analyzed using FlowJo v10. Compensation and instrument settings were standardized across all samples to ensure comparability of MFI measurements. For in vitro staining of PD-L1 and PD-L2, B16-PD-L2 or TS/A cells were detached from culture flasks, washed with PBS containing 0.5% BSA, and 0.5 × 10⁶ cells were stained with anti-mouse PD-L1-PE, PD-L2-PE or control isotype antibodies. To assess the binding of 27907 to tumor cells, 0.5 × 10⁶ cells were incubated with 2 µg of 27907 for 30 min on ice, followed by washing and secondary staining with an Alexa Fluor488-conjugated anti-human IgG antibody. Samples were acquired using a BD FACSVerse flow cytometer or NovoCyte flow cytometer and analyzed with FlowJo software.

### IHC analysis

Tumors were fixed in 10% neutral buffered formalin and embedded in paraffin; slides were sectioned at 5-µm thickness and stained with Hematoxylin (Bio-Optica) and Eosin (Bio-Optica) for histological examination. Histological evaluation was performed using light microscopy to assess tumor architecture, viability, and the presence of necrotic regions. Necrosis was defined as areas of eosinophilic, anuclear tissue, often with ghost outlines of cells and surrounding inflammatory infiltrates. Digital images were captured using a (Nikon Eclipse Ci-L) and quantified. For immunohistochemical evaluation, slides were deparaffinized, serially rehydrated and, after the antigen retrieval procedure (microwave citrate buffer, pH 6.0, 10 min), incubated with the following primary antibodies: rabbit anti-mouse F4-80 antibody (70076, Cell Signalling), rabbit anti-mouse CD3 antibody (ab16669, Abcam), and anti-mouse Foxp3 (BioLegend, Cat#126403), according to the manufacturer’s protocols, followed by the appropriate secondary antibodies (Jackson Laboratories). Immunoreactive antigens were detected using streptavidin peroxidase (Thermo Scientific) and the DAB Chromogen System (Dako). After chromogen incubation, slides were counterstained in Hematoxylin (Bio-Optica) and images were scanned with Nanozoomer scanner from Hamamatsu. The percentage of CD3, Foxp3 and F4-80 positive cells was evaluated on whole tumor section (*n* = 2–3 samples per group) and analyzed with Qu-Path 0.3.2 software using positive cell detection tool. Statistical differences between the experimental groups were evaluated by applying unpaired Student’s t-test. Differences were statistically significant when *p* values were less than 0.05. The percentage of positive cells was plotted using the GraphPad Prism10 software (GraphPad).

For IF, slides were incubated overnight with the following antibodies: rabbit anti-mouse Caspase3 antibody (af835, R&D System), rabbit anti-mouse CD31 antibody (ab182981, Abcam) mixed with rabbit anti-mouse CD105 antibody (ab221675, Abcam) and rabbit anti-CD62p antibody (ab255882, Abcam), anti-mouse CD206 (BioLegend, Cat#141705) and F4/80 (clone: BM8). IF was developed using TSA Plus Cyanine 3 Systems (Cat#NEL744001KT, Akoya Biosciences) and goat anti-rabbit Alexa Fluor 488 (Cat#A11008, ThermoFisher) as secondary antibodies; nuclei were stained with DAPI (Sigma). Image acquisition was performed using Zeiss LSM800 confocal microscope. The number of apoptotic endothelial cells was counted in digital images and divided by the corresponding total vascular area, measured in pixels, using Adobe Photoshop. Quantitative analysis was performed on 1–3 × 200 microscopic fields per sample.

### Statistical analysis

Statistical significance was evaluated using GraphPad10 software. Differences in mouse survival were analyzed using Mantel-Cox log-rank test. Differences in tumor growth were analyzed using two-way ANOVA. Differences in cell viability, IHC, IF and FACS data were analyzed using two-tailed unpaired Student’s t-test or ordinary one-way ANOVA. A threshold of *p*-value < 0.05 was considered statistically significant. Asterisks indicate levels of significance as follows: * *p* < 0.05; ** *p* < 0.01; *** *p* < 0.001; **** *p* < 0.0001. The number of technical or biological replicates of the experiments described and the specific statistical test used, are reported in the corresponding figure legends.

## Results

### 8803 potently activates the STING pathway in reporter and tumor cell lines

**Fig. 1:**
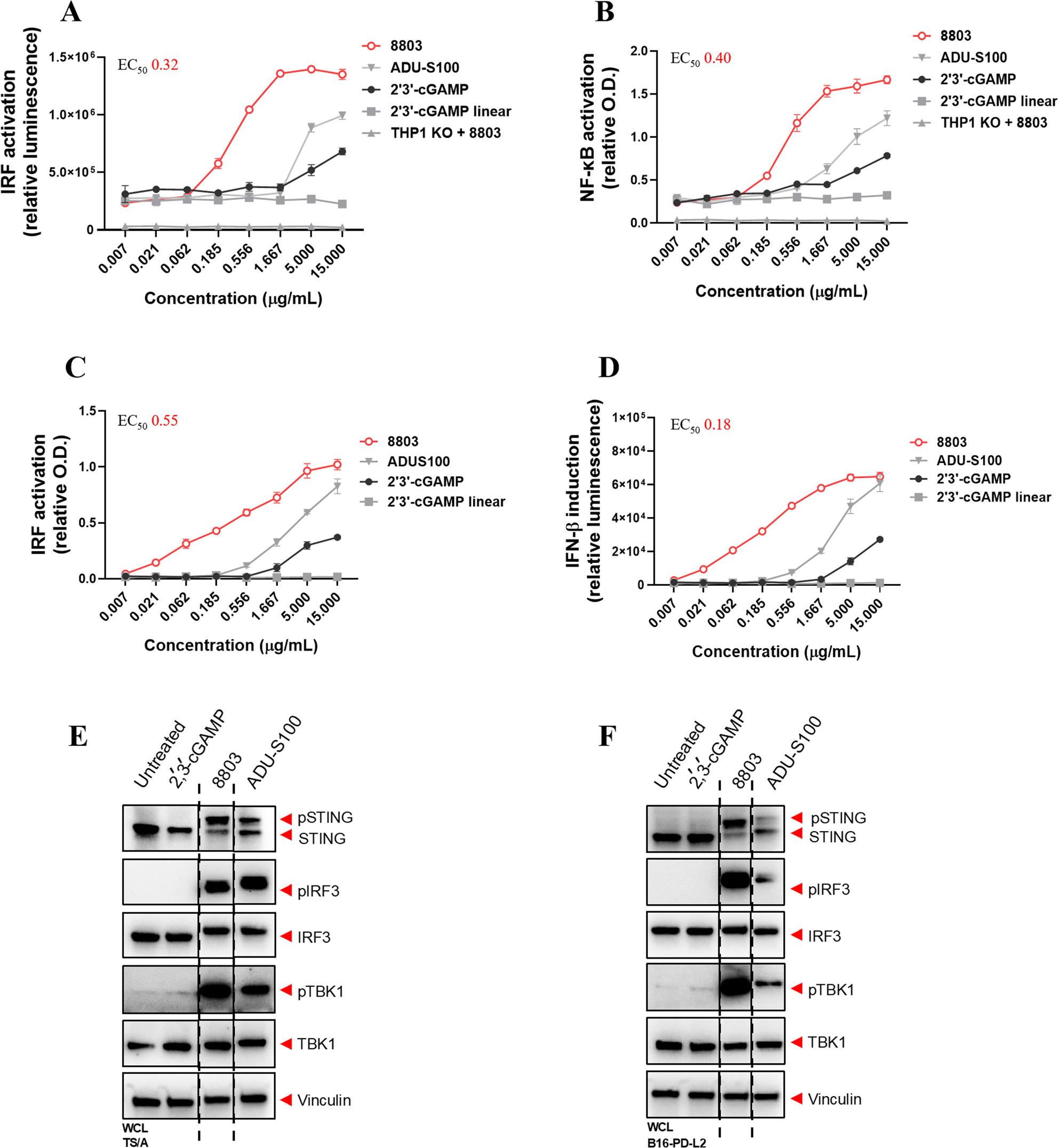
8803 induces a strong activation of the STING pathway. In vitro potency bioassays were performed using THP1-Dual™ (**A**, **B**) and 293-Dual™ (**C**, **D**) reporter cell lines, expressing human and mouse STING, respectively, to compare 8803 with the commercially available CDNs 2', 3'-cGAMP, ADU-S100, and a linear inactive 2', 3'-cGAMP (negative control) in a dose-response manner. Results are shown for activation of IRF (IRF3 /IRF7) (**A**, **C**)and NF-κB (**B**), and for IFN-β induction (**D**). In (**A**, **B**), THP1 STING knockout (THP1 KO) cells were included as a negative control to confirm pathway specificity. **E**, **F** Western blot analysis of STING pathway activation in B16-PD-L2 (**E**) and TS/A (**F**) cancer cell lines upon treatment with different STING agonists. Vinculin was used as a loading control. EC_50_is reported as µg/mL

To evaluate the effector mechanisms and potency of the STING agonist 8803 and the dual anti-PD-L1/PD-L2 antibody 27907, we first tested them individually in vitro. The ability of 8803 to activate both human and mouse STING was compared with other known STING agonists using THP1-Dual™ and 293-Dual™ reporter cell lines, expressing human and mouse STING, respectively. As shown in Fig. 1A–D, 8803 demonstrated EC₅₀ values of approximately 0.32/0.40 µg/mL in THP1-Dual™ cells; it was approximately 52-fold more potent than the endogenously produced ligand 2’3’-cGAMP and 20-fold more potent than ADU-S100 in activating both IRF (IRF3/IRF7) and NF-κB pathways. STING pathway specificity was confirmed using THP1-KO cells as negative controls (Fig. 1A, B). Similar potency was observed for IRFs activation and IFN-β induction in 293-Dual™ reporter assay, with EC₅₀ values of approximately 0.55/0.18 µg/mL, respectively (Fig. 1C, D).

To investigate whether 8803 could directly activate the STING pathway in tumor cells, B16-PD-L2 [26] and TS/A [27, 28] cells were treated with 10 µg/mL of 8803, 2’3’-cGAMP, or ADU-S100 for 1 h, followed by Western blot analysis. 8803 robustly activated the STING pathway compared to commercial agonists, as evidenced by phosphorylation of STING, TBK1, and IRF3 (Fig. 1E, F). To assess whether 8803 exerted direct cytotoxic effects on tumor cells, B16-PD-L2 and TS/A cells were treated with 10 µg/mL of 8803 for 48 h. No overt changes in morphology were observed, and MTT assays confirmed no significant reduction in viability (Supplementary Fig. 1A, B). These results suggest that the anti-tumor effects of 8803 in vivo are unlikely to be mediated by direct cytostatic or cytotoxic actions on tumor cells.

### 27907 potently blocks the PD-1 pathway, binds FcγRs, and mediates ADCC and ADCP

**Fig. 2:**
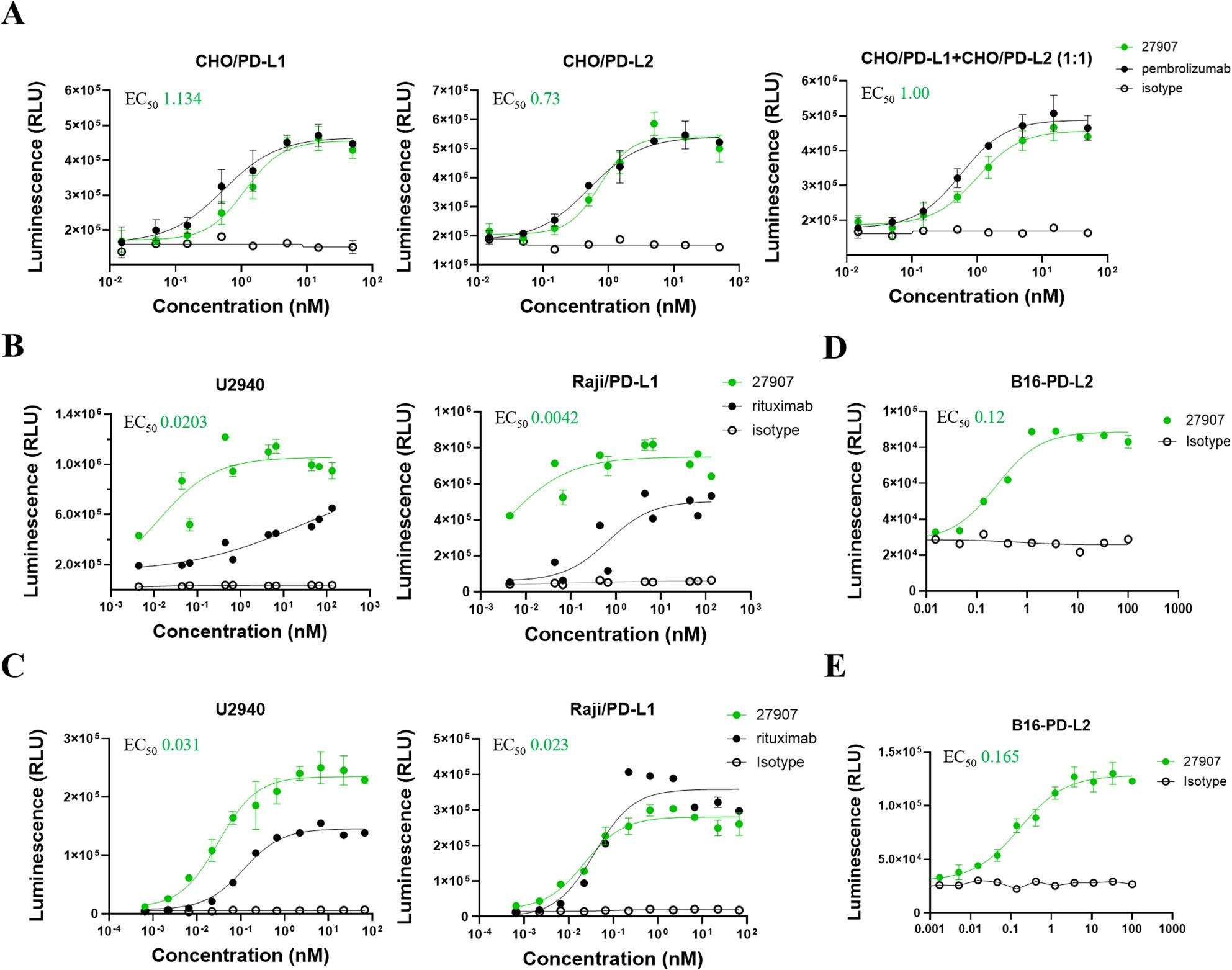
27907 dual specific antibody inhibits PD-1 binding to PD-L1/PD-L2 and engages Fc𝛾Rs. **A** Dose-dependent inhibition of PD-1 binding to PD-L1/PD-L2 expressed on CHO cells by 27907 with the human IgG1 heavy chain constant region containing GASDIE mutations using the Promega PD-1/PD-L1 and the PD-1/PD-L2 blockade bioassays. **B**, **C** Dose-dependent induction of ADCC (**B**) and ADCP (**C**) activities by 27907 against human B cell lines using Promega reporter bioassays with NFAT-luciferase reporter Jurkat T cells expressing Fc𝛾RIIIa (for ADCC) and Fc𝛾RIIa-H (for ADCP). The EC₅₀ of 27907 values for ADCC and ADCP induction ranged between 0.004 – 0.031 nM, indicating potent Fc-mediated effector function. Pembrolizumab and rituximab were included as positive controls, while the isotype control served as negative control. **D** ADCC and (**E**) ADCP activities induced by 27907 or isotype control against B16-PD-L2 murine tumor cells, with EC₅₀ of 27907 values for ADCC and ADCP 0.12 and 0.165 nM, respectively. A higher luminescence signal, expressed as Relative Luminescence Units (RLU), indicates an efficient ADCC or ADCP activation pathway. Mean ± SEM (*n* = 3) is displayed for each concentration of each antibody. **A**-**E** Nonlinear regression was used to generate best-fit curves in GraphPad Prism. EC_50_ is reported as nM.

Binding kinetics of 27907 to human and mouse PD-L1 and PD-L2 were measured by bio-layer interferometry. 27907 bound human and mouse PD-L1 and PD-L2 with comparable high affinity (human PD-L1, 5.32 × 10⁻¹⁰ M; human PD-L2, 5.60 × 10⁻¹⁰ M; mouse PD-L1, 5.44 × 10⁻¹⁰ M; mouse PD-L2, 5.69 × 10⁻¹⁰ M). The ability of 27907 to interrupt the PD-1/PD-L1 and PD-1/PD-L2 inhibitory pathways was first analyzed using CHO/PD-L1 and CHO/PD-L2 cells co-cultured with Jurkat T cells stably expressing human PD-1 and an NFAT-inducible luciferase reporter. In both PD-L1 and PD-L2 settings, 27907 effectively prevented PD-1-mediated inhibition of Jurkat T cell activation, displaying comparable potency to pembrolizumab, with EC₅₀ values of approximately 0.73–1.1 nM (Fig. 2A). To assess the binding of 27907 on mouse PD-L1 and PD-L2 on the cell surface, we used the B16-PD-L2 and 293/msPD-L2 cells. Both cell lines express murine PD-L2, but while B16-PD-L2 cells also express murine PD-L1, 293/msPD-L2 are PD-L1 negative (Supplementary Fig. 2A). Binding of 27907 to B16-PD-L2 and parental B16F10 was confirmed by FACS (Supplementary Fig. 2B).

To evaluate the FcγR–mediated effector functions of 27907 in human tumor cell lines expressing PD-L1 and/or PD-L2, we selected tumor B-cell lines as target cells due to their clinical relevance, high surface expression of B-cell markers such as CD20, and compatibility with Fc-mediated cytotoxicity assays. Specifically, Raji-PD-L1 and U2940 cells provide two complementary PD-L1/PD-L2 expression contexts. Raji cells, which do naturally express low levels of PD-L1 and no PD-L2, were engineered to express PD-L1 exogenously (Raji/PD-L1), allowing precise control over target antigen density and specificity of 27907 binding. In contrast, U2940 cells endogenously express high levels of both PD-L1 and PD-L2, providing a more physiologically relevant model that reflects native antigen presentation in certain lymphomas [24–26]. Effector cells were Jurkat reporter cells expressing FcγRIIIa or FcγRIIa-H, enabling luciferase-based detection of FcγR engagement and a robust assessment of the ability of 27907 to induce both ADCC and ADCP. Of note, 27907 induced superior ADCC (Fig. 2B) and comparable ADCP in Raji/PD-L1 cells (Fig. 2C) as compared to rituximab (anti-CD20), which was used as a positive control to confirm pathway activation. We next evaluated the binding of 27907 to murine FcγRs to support its functional assessment in mouse models. ELISA plates were coated with immobilized 27907, 27907-LALA-PG, or 27907-IgG2a mouse (27907-mIgG2a) isotypes (30–0.12 µg/mL), followed by incubation with biotinylated mouse FcγRs. 27907 demonstrated binding to mouse FcγRIII (CD16) and FcγRIIb (CD32b) comparable to that observed with the murine IgG2a isotype variant, 27907-mIgG2a (Supplementary Fig. 2C, D). These findings are consistent with established species-specific FcγR–IgG interaction profiles and support the suitability of this antibody format for evaluation in murine in vivo studies [32–35]. Functional activity was then assessed using B16-PD-L2 target cells in Promega ADCC and ADCP reporter assays. 27907 induced robust, dose-dependent activation of both pathways, whereas the isotype control showed minimal activity (Fig. 2D, E). To confirm that 27907 effector functions are mediated through FcγR engagement, its activity was compared with that of 27907-LALA-PG isotype variant (Supplementary Fig. 3A). As expected, this isotype variant exhibited no activity in the biological reporter assays. (Supplementary Fig. 3B-F).

### Combination treatment with 8803 and 27907 controls tumor growth and prolongs survival in B16-PD-L2 melanoma and ICB-resistant TS/A mammary tumor models

First, we evaluated the potential binding competition between therapeutic antibody 27907 and staining antibodies using the B16-PD-L2 cells. FACS analysis showed no evidence of binding competition, indicating that 27907 recognizes epitopes distinct from those targeted by the staining antibodies. In addition, PD-L1 and PD-L2 surface expression levels in B16-PD-L2 cells remained unchanged following 24-hour treatment with 27907 (Supplementary Fig. 4A). Then, C57BL/6 mice were implanted s.c. with 1 × 10⁵ B16-PD-L2 cells, left untreated and euthanized when the tumor reached 100–200 mm³. B16-PD-L2 tumors were dissociated and analyzed by FACS, demonstrating sustained PD-L1 and PD-L2 expression on tumor and stromal (CD45⁻) cells (Supplementary Fig. 4B) and immune (CD45⁺) infiltrates (Supplementary Fig. 4C, D). Notably, PD-L2 expression was high on CD45^−^ cells, while it was limited on immune cells, present on approximately 17% of dendritic cells (DCs) and 7% of TAMs.

**Fig. 3:**
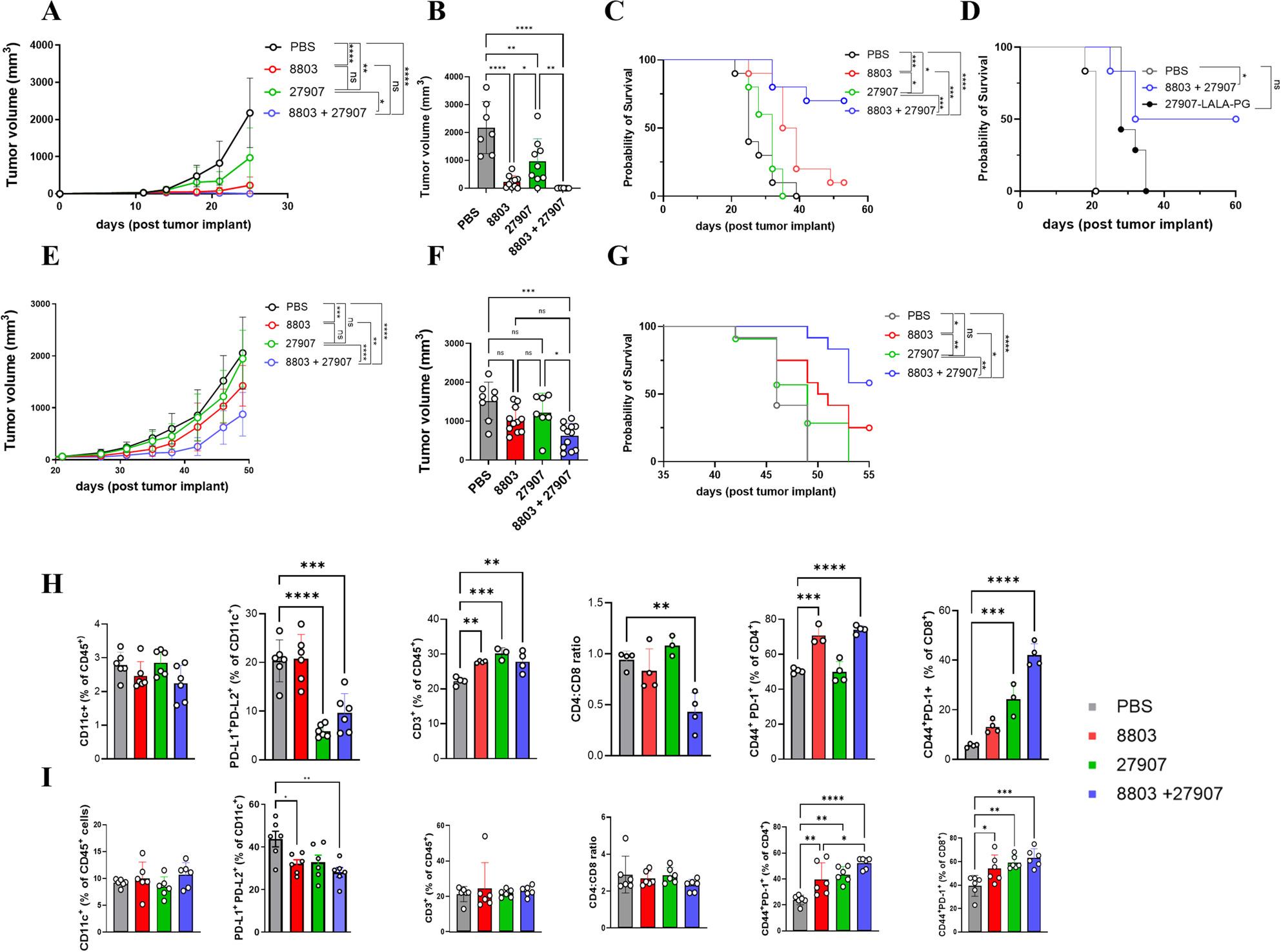
The combination of 27907 and 8803 enhances antitumor responses in both the B16-PD-L2 melanoma and TS/A mammary tumor models. **A**–**D** B16-PD-L2 melanoma model. C57BL/6 mice were injected subcutaneously (s.c.) in the left flank with 1 × 10⁵ B16-PD-L2 tumor cells. **A**-**C** Upon tumors reaching 75 – 100 mm³, mice were randomized into four treatment groups (PBS, 27907, 8803, or combinations; *n* = 10–12 mice per group) and received two i.t. injections of either PBS or 10 µg of 8803 at 3–4-day intervals, and i.p. injections twice weekly for three weeks with either PBS or 200 µg of 27907. **A** Tumor growth curves over time, (**B**) tumor volumes of individual mice at 21 days post-implant, and (**C**) overall survival of mice treated with 27907, 8803, or the combination. **D** Overall survival of B16-PD-L2 tumor bearing C57BL/6 mice (*n* = 6-7 mice per group) treated with 8803 (10 µg/dose, i.t. twice at 3–4-day interval) in combination with either 27907 or the Fc-silent variant 27907-LALA-PG (400 µg/dose, i.p. twice weekly for three weeks) initiated when tumors reached an average volume of 110 mm³. **E**-**G** TS/A mammary tumor model. BALB/c mice were injected orthotopically with 1 × 10⁵ TS/A cells into the fourth left mammary gland. When tumors reached 75–100 mm³, mice were randomized into treatment groups (*n* = 10–12 mice per group) and received two i.t. injections of either PBS or 10 µg of 8803 at 3–4-day intervals, and i.p. injections twice weekly for three weeks with either PBS or 200 µg of 27907. **E** Tumor growth curves, (**F**) tumor volume in individual mice at 46 days post-implant, and (**G**) overall survival. **H**, **I** FACS analysis of spleens from mice bearing (**H**) B16-PD-L2 or (**I**) TS/A tumors sacrificed 3–4 days after treatment completion. Data are presented as mean ± SEM. Statistical significance was determined by ordinary one-way ANOVA, with *P < 0.05, **P < 0.01, ***P < 0.001, and ***P < 0.0001

To evaluate therapeutic efficacy, C57BL/6 mice implanted s.c. with 1 × 10⁵ B16-PD-L2 cells once tumors reached 75–100 mm³, were randomized into four treatment groups: 27907 i.p., 8803 i.t., the combination, and PBS control (i.p. and i.t.). Monotherapy with 27907 modestly inhibited tumor growth, while 8803 alone resulted in substantial tumor suppression (Fig. 3A, B). The combination therapy, however, led to rapid tumor regression, evident as early as day 3 from the first treatment (Fig. 3A), with marked reductions in tumor volume at day 21 (Fig. 3B). Importantly, the combination also resulted in significantly prolonged survival as compared to the single treatments, with 66.7% (6/9) of mice remaining tumor-free until the study endpoint on day 55 (Fig. 3C). To assess whether the survival benefit observed with the combination treatment was influenced by GASDIE-enhanced effector Fc functions of 27907, mice bearing B16-PD-L2 tumors at an average volume of 110 mm^3^ were treated with 8803 in combination with either 27907-GASDIE or the Fc-silenced variant 27907-LALA-PG. A survival benefit was observed with the combination including the GASDIE variant with 50% tumor-free mice at day 60 after tumor challenge versus 0% survival in the combination with the LALA-PG variant treated group (Fig. 3D). These results support a contribution of FcγR engagement to the therapeutic activity of 27907.

To assess efficacy in an ICB-resistant setting, we used the Her2⁺ TS/A mammary carcinoma model [27, 28]. FACS analysis revealed low PD-L1 and no PD-L2 expression on TS/A cells from in vitro cultures (Supplementary Fig. 5A). To evaluate expression in vivo, TS/A cells were orthotopically implanted into the mammary fat pad of BALB/c mice. Upon reaching 100–200 mm³, tumors were dissociated and analyzed by FACS. PD-L1 was detected on all CD11b⁺Ly6C^high^ mMDSCs, ~ 5% of CD11b⁺Ly6G^high^ gMDSCs, 36% of TAMs, and ~ 27% of CD45⁻ stromal/tumor cells (Supplementary Fig. 5B, C). PD-L2, in contrast, was highly expressed on mMDSCs, gMDSCs, and TAMs, but absent from CD45⁻ tumor/stromal cells (Supplementary Fig. 5B, C), indicating this model enables evaluation of 27907’s effects on the immune compartment rather than on tumor cells directly. For treatment, BALB/c mice bearing 75–100 mm³ TS/A tumors were randomized into the same four treatment groups as for the B16-PD-L2 model. Both monotherapies modestly inhibited tumor growth, with 8803 showing a more pronounced effect and extended survival (Fig. 3E-G). These effects were further enhanced in the combination group, where tumor volumes were significantly reduced by day 46 (Fig. 3F), and 58% of mice remained below the survival threshold (1,500 mm³) by day 55, compared to 25% in the 8803-monotherapy group (Fig. 3G).

To investigate the mechanisms underlying the observed anti-tumor effects, a separate cohort of B16-PD-L2 and TS/A tumor-bearing mice was sacrificed 3–4 days post-treatment for immunophenotyping of spleen cells. FACS analysis revealed reduced frequencies of CD11c⁺PD-L2⁺PD-L1⁺ DCs, likely corresponding to mregDCs [10, 13, 14], in the 27907-treated group of the B16-PD-L2 model, in the 8803-treated group of the TS/A model, and in the combination treatment groups in both models (Fig. 3H, I). Concurrently, the frequency of CD3⁺ T cells increased in all treatment groups in the B16-PD-L2 model. Notably, CD44⁺PD-1⁺CD4⁺ and CD8⁺ T cells were significantly elevated in both models, with the most robust responses observed following combination therapy (Fig. 3H, I), suggesting enhanced T cell activation and effector differentiation.

### Combined 8803 and 27907 treatment enhances T cell infiltration and reduces immunosuppressive myeloid cells at the tumor site

**Fig. 4:**
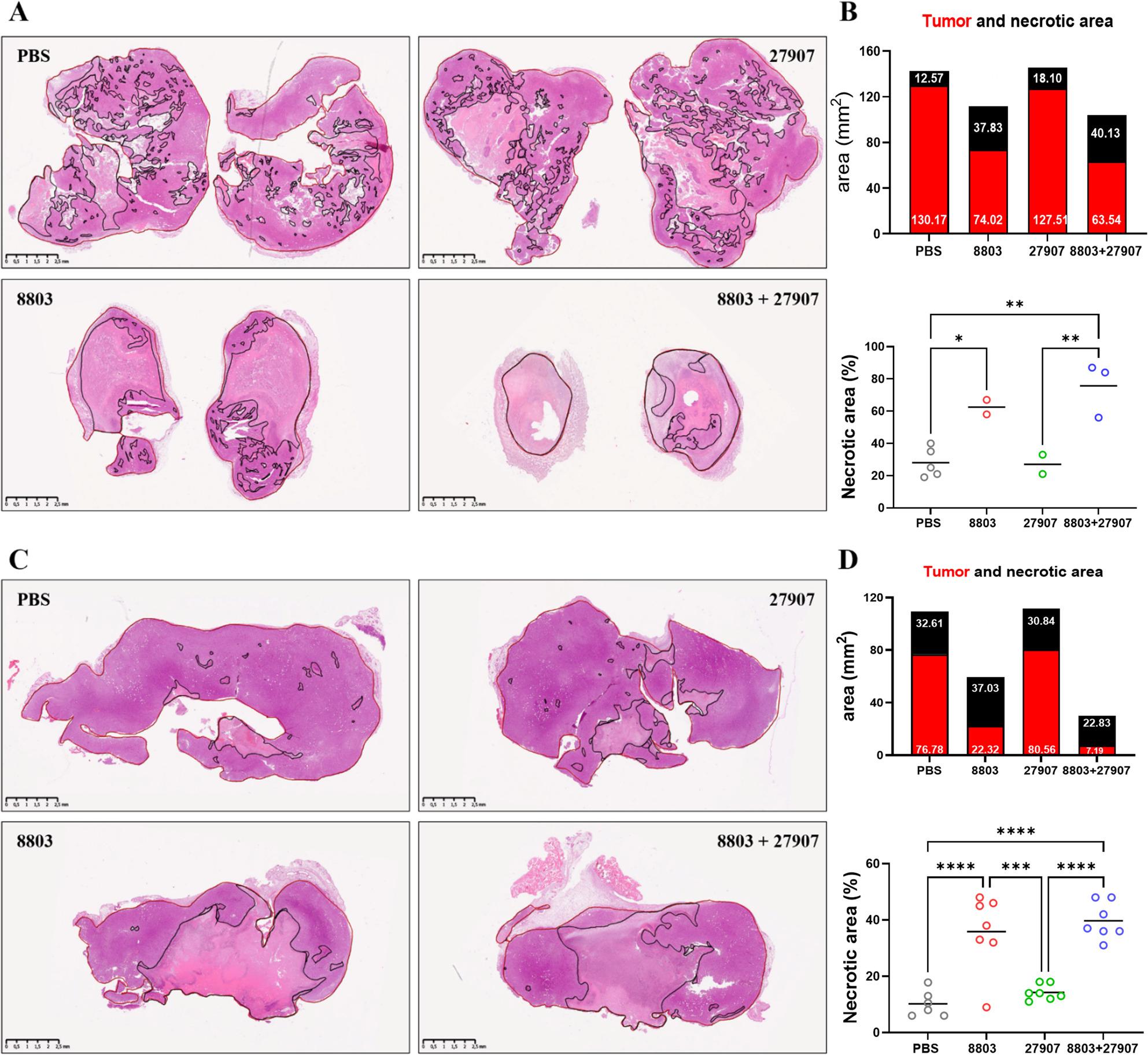
Histological analysis of the tumors revealed extensive necrotic areas in 8803 and combination-treated mice. **A** Representative H&E-stained images of B16-PD-L2 tumor sections from each treatment group, with necrotic areas highlighted. **B** Histograms and dot plots showing tumor volume and percentage of necrotic areas in each treatment group. **C** Representative H&E-stained TS/A tumor sections from each group, with necrotic regions highlighted. **D** Histograms and dot plots quantifying tumor volume and necrosis across treatment conditions. Statistical analyses were performed using GraphPad Prism software. Significance was assessed using (**B**, **D**) ordinary one-way ANOVA, with *P < 0.05, **P < 0.01, ***P < 0.001, and ***P < 0.0001

**Fig. 5:**
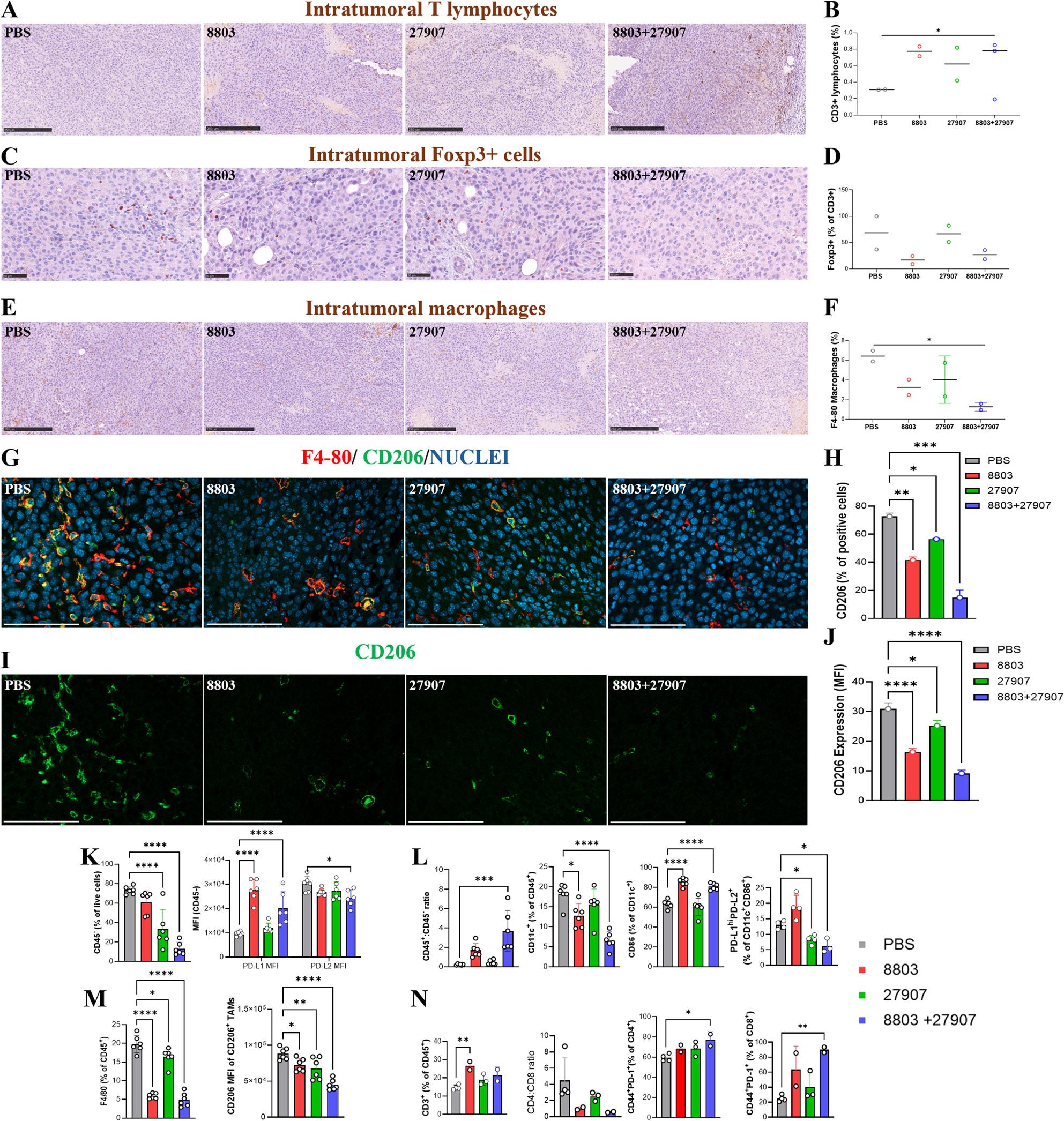
Combination of 27907 and 8803 alters the TME in B16-PD-L2 melanoma model. C57BL/6 mice were implanted s.c. with 1 × 10⁵ B16-PD-L2 melanoma cells. Treatment with 27907 (200 µg per dose, i.p.) or PBS were administered on days 11, 14, 18, and 21. The STING agonist 8803 (10 µg per dose, i.t.) was delivered on days 11 and 14. Tumors were harvested on day 17 from 2–3 mice per group and processed for TME analysis. **A**-**F** IHC images of tumor sections were stained with anti-CD3 (T cells), anti-Foxp3 (Tregs), and anti-F4/80 (macrophages). Representative IHC images (magnification x200) and corresponding quantifications are shown for (**A**, **B**) CD3⁺ T cells, (**C**, **D**) Foxp3⁺ Tregs, and (**E**, **F**) F4/80⁺ macrophages. **G**-**J** IF analysis of tumors stained with anti-F4/80, anti-CD206 (M2 macrophage marker), and DAPI (nuclei) is shown as representative images and fluorescence quantification of F4/80⁺CD206⁺ macrophages. **K**-**N** FACS analysis was performed 3–4-days after the final treatment to assess immune, tumor, and stromal cell populations within B16-PD-L2 tumors. **K** Frequency of CD45^-^ tumor/stromal cells and analysis of PD-L1 and PD-L2 expression on live CD45⁻ cells, including both tumor and stromal compartments. **L** CD45⁺ immune cell infiltration was assessed, including analysis of the CCD45⁺/CD45^-^cell ratio, CD11c⁺ DCs, activated DCs (CD86⁺CD11c⁺), and co-expression of PD-L1 and PD-L2 on CD11c⁺CD86⁺ subsets, including PD-L1^hi^PD-L2⁺ populations. **M** TAMs, identified as F4/80⁺ cells, and their expression of CD206 were analyzed and quantified. **N** T cell populations were assessed, including total CD3⁺ T cells, CD4⁺/CD8⁺ ratios, and activated T cell subsets (CD44⁺PD-1⁺) within both CD4⁺ and CD8⁺ compartments. Data are presented as mean ± SEM. Statistical significance was determined by ordinary one-way ANOVA, with *P < 0.05, **P < 0.01, ***P < 0.001, and ***P < 0.0001

To further assess the impact of 8803 and 27907 at the tumor site, histological analysis and IHC were performed. B16-PD-L2 tumors from 8803-treated mice exhibited extensive necrotic regions compared to those in the control group, indicating substantial tumor cell death. These necrotic areas appeared more extensive in tumors from the combination therapy group, consistent with the enhanced immune activation observed systemically (Fig. 4A, B). Similarly, TS/A tumors from 8803-treated mice displayed prominent necrotic regions, with the greatest extent of necrosis observed following combination treatment (Fig. 4C, D). IHC revealed that the TME was predominantly myeloid-rich, with limited lymphoid infiltration in both B16-PD-L2 (Fig. 5) and TS/A (Supplementary Fig. 6) models. Treatment with 27907 alone had minimal impact on immune cell composition. In contrast, 8803, particularly when combined with 27907, significantly increased T cell infiltration, as indicated by increased CD3⁺ cell density (Fig. 5A, B; Supplementary Fig. 6A, B). 8803 and combination treatment groups showed reduced Foxp3⁺ Tregs, suggesting a shift away from immunosuppression (Fig. 5C, D and Supplementary Fig. 6C, D). The overall number of intratumor TAMs was significantly reduced in the B16-PD-L2 model, with the largest decrease observed in the combination group (Fig. 5E, F), while remaining substantially unchanged in the TS/A model (Supplementary Fig. 6E, F). IF analysis showed that CD206⁺ M2-like TAMs were markedly diminished in number and staining intensity following the combination therapy in both tumor models (Fig. 5G-J and Supplementary Fig. 6G-J), consistent with a potential reprogramming of the TAM compartment toward a less immunosuppressive phenotype. These findings are consistent with a model in which STING activation and PD-1 blockade act cooperatively to reprogram the immune microenvironment, reduce immunosuppressive populations, and promote robust T cell-mediated anti-tumor activity.

To further characterize the TME modulation induced by 8803 and 27907, FACS analysis was conducted on B16-PD-L2 tumor tissues. Both 27907 monotherapy and the combination treatment led to a substantial reduction in tumor and stromal (CD45⁻/live) cells, suggesting cytotoxic effects beyond immune cell compartments. Interestingly, while PD-L2 expression on CD45⁻ cells remained unchanged, PD-L1 expression was upregulated, likely reflecting a compensatory immune resistance mechanism (Fig. 5K). In contrast, CD45⁺ immune cells were increased in all treatment groups, indicating enhanced recruitment or retention of leukocytes within the tumor. Despite a reduction in the frequency of CD11c⁺ cells, their activation status was markedly improved, as shown by elevated expression of the co-stimulatory molecule CD86, that was significant in the 8803 and combination groups, suggestive of an improved antigen presentation and priming capacity. Importantly, a decrease in the frequency of PD-L1⁺PD-L2⁺CD86⁺CD11c⁺ DCs, the characteristic phenotype of mregDCs, was observed in both 27907 and combination groups (Fig. 5L), suggesting relief from tolerogenic programming.

Similarly, both single treatments and the combination induced a significant reduction in TAMs and their immunosuppressive phenotype, as highlighted by a decreased expression of the M2 marker CD206 (Fig. 5M). Further immunophenotyping revealed increased PD-L1 expression across multiple innate immune subsets, including mMDSCs and gMDSCs, TAMs, and DCs, both in the TME and in the TDLN (Supplementary Fig. 7A-C). In contrast, PD-L2 expression was more selective, predominantly restricted to TAMs and DCs, and remained consistently lower than PD-L1 across all immune compartments of the TME (Supplementary Fig. 7A-C).

The adaptive immune compartment was also modulated, with increased frequencies of CD3⁺ T cells observed across all treatment groups, particularly in the presence of 8803. Notably, a reduction in the CD4^+^/CD8^+^ T cell ratio was observed, driven by an enrichment of CD44⁺PD-1⁺CD8⁺ T cells, a phenotype associated with effector and memory-like activation. These effects were most pronounced following the combination therapy (Fig. 5N).

### Immune remodeling and platelet-mediated vascular damage underlie treatment-induced necrosis in melanoma and breast tumors

**Fig. 6:**
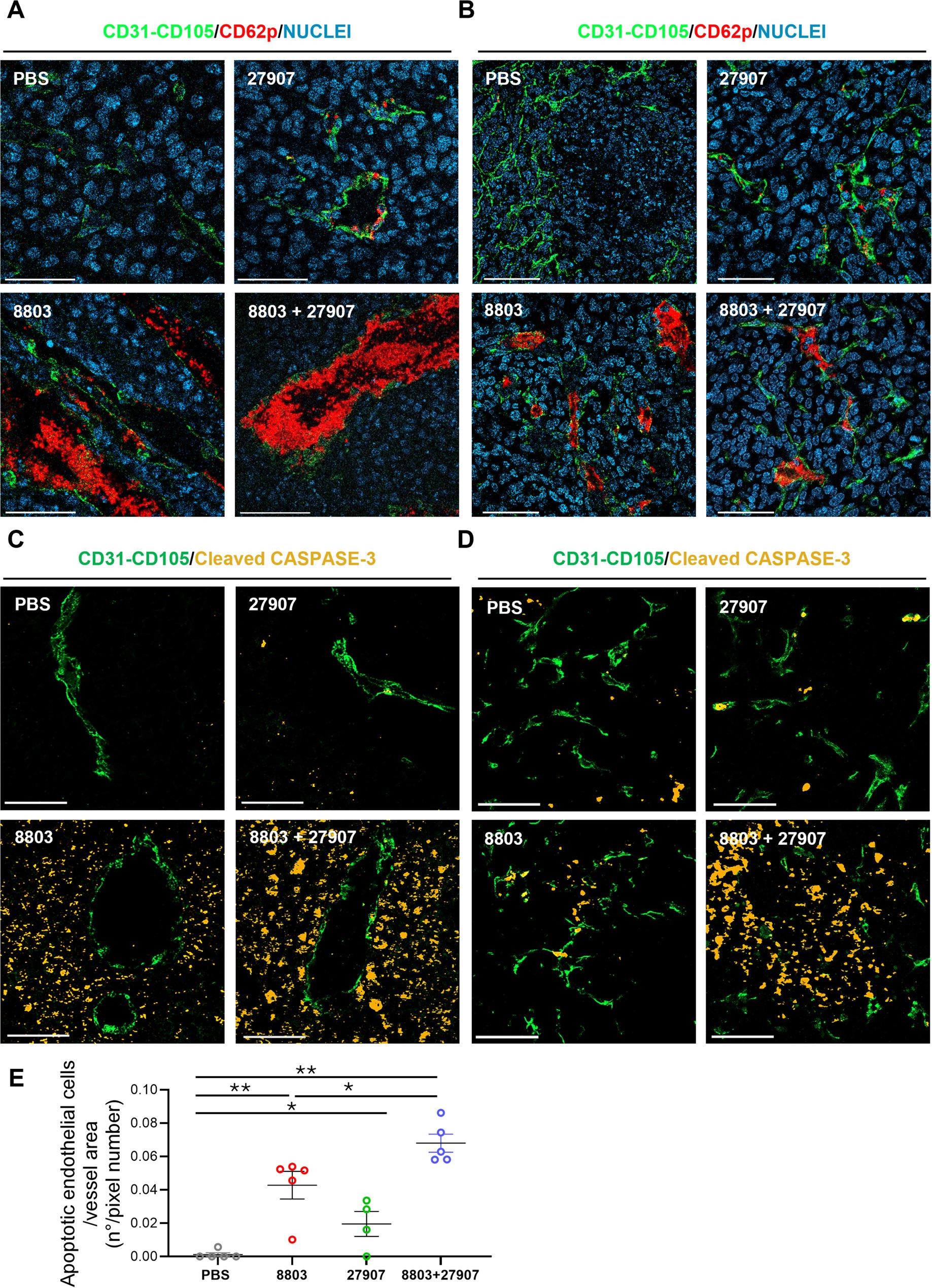
8803 and 27907 treatment induces platelet aggregation, blood vessel injury, and apoptosis in B16-PD-L2 and TS/A tumor models. Mice bearing B16-PD-L2 or TS/A tumors were treated with 8803, 27907, or their combination, as described in the Materials and Methods. Tumors were harvested 3–4 days after the final treatment and processed for IF analysis. Representative images show CD62p (P-selectin, red) expression in B16-PD-L2 (**A**) and TS/A (**B**) tumors (Magnification x400). Strong platelet aggregation and CD62p endothelial cell expression were observed following treatment with 8803 and the combination therapy. In contrast, CD62p expression was minimal in untreated tumors, while 27907 treatment modestly increased CD62p expression in proximity to blood vessels (marked by CD31/CD105, green). Representative images show caspase-3 (yellow) expression in endothelial cells marked by CD31/CD105 (green) in B16-PD-L2 (**C**) and TS/A (**D**) tumors (Magnification x400). The quantitative evaluation of apoptotic endothelial cells divided by the total endothelial area is shown for TS/A samples (**E**). Due to extensive necrosis in B16-PD-L2 tumors treated with 8803 alone or in combination, quantitative analysis was not performed, as tissue integrity was compromised and endothelial structures could not be reliably identified. Statistical significance was determined by ordinary one-way ANOVA, with *P < 0.05, **P < 0.01, ***P < 0.001, and ***P < 0.0001

To gain deeper insight into the mechanistic causes of the extensive necrotic areas observed in the histological analysis, we examined CD62p (P-selectin) expression, a marker of platelet activation and endothelial injury (Fig. 6). Baseline CD62p expression was minimal in tumor vasculature, while 27907 alone modestly increased CD62p on endothelial cells. Treatment with 8803 strongly induced CD62p on activated and aggregated platelets, indicating robust platelet activation and thrombus formation. Combination therapy preserved 8803’s thrombotic effects and further enhanced endothelial CD62p expression, as demonstrated by co-localization with the endothelial markers CD31/CD105 in B16-PD-L2 (Fig. 6A**)** and TS/A (Fig. 6B**)** tumors. Given that PD-L1 is expressed on endothelial cells [15], we assessed whether 8803 with or without 27907 affected endothelial cell viability. Activated caspase-3 staining showed no apoptotic endothelial cells in untreated tumors, whereas a few apoptotic cells were seen in the endothelial cells of 27907 tumors. Interestingly, in 8803-treated B16-PD-L2 tumors, activated caspase-3-stained cells were only found among tumor and stromal cells, whereas in the combination treatment, an increased number of endothelial and tumor/stromal cells were stained (Fig. 6C). A similar pattern was observed in the TS/A breast carcinoma model, where 27907 treatment induced a slight but evident endothelial apoptosis which was further increased in the presence of 8803 (Fig. 6D, E).

### 8803 Activates STING signaling and upregulates PD-L1 in mouse endothelial cells, enhancing sensitivity to 27907

**Fig. 7:**
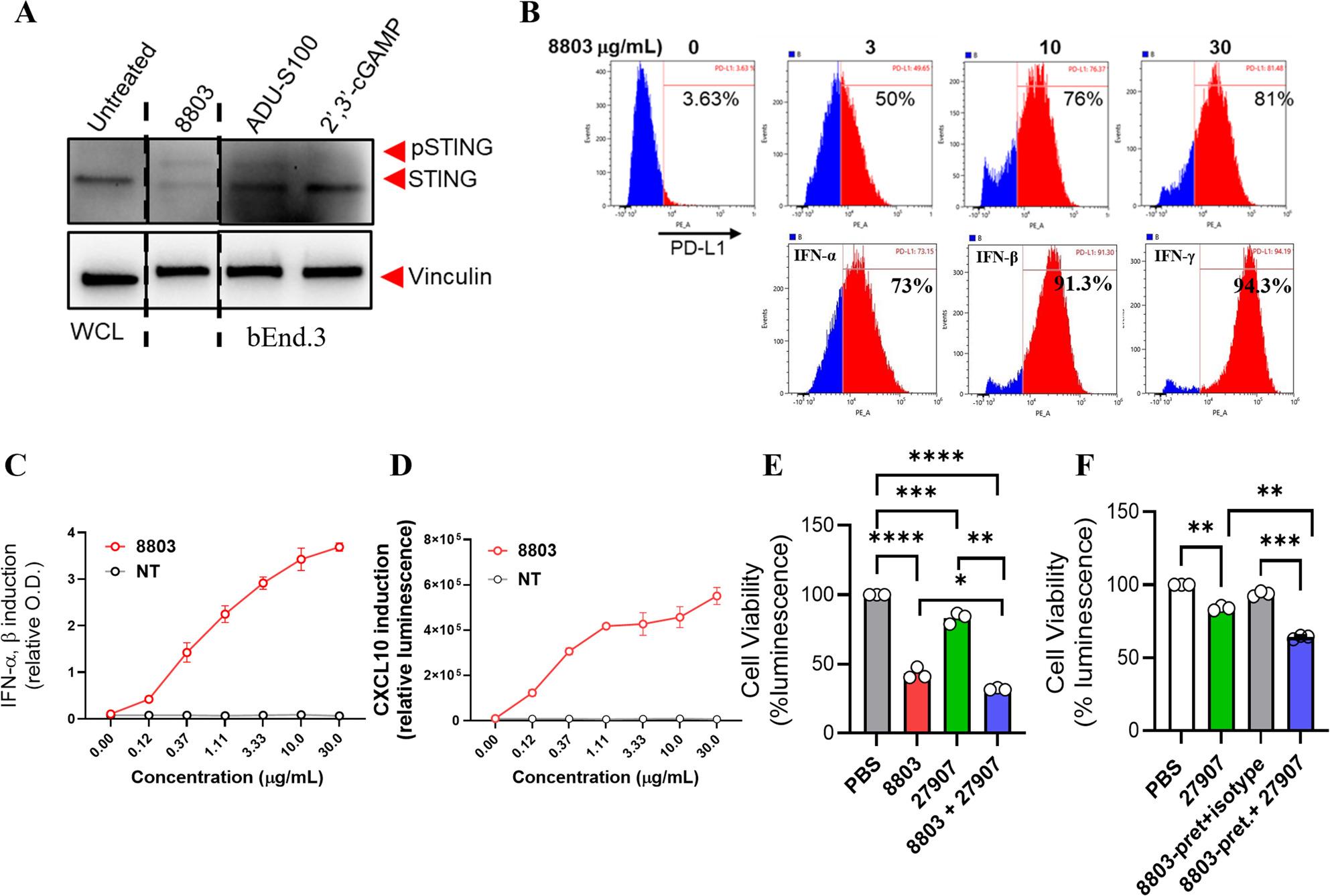
8803 induces activation of the STING pathway in endothelial cells, upregulates PD-L1 expression and enhances immune-mediated cytotoxicity. **A** STING pathway activation in mouse endothelial bEnd.3 cells demonstrated by phosphorylation of STING via western blot following treatment with 8803 or commercial CDNs. **B** Surface expression of PD-L1 on bEnd.3 cells after treatment with 8803, recombinant IFN-α, IFN-β, or IFN-γ analyzed by FACS. **C**, **D** Induction of cytokine production in bEnd.3 cells treated with 8803, assessed via supernatant analysis using cytokine reporter cell lines. **E** Cytotoxicity assay showing increased killing of bEnd.3 cells co-cultured with activated splenocytes (pre-treated with mouse GM-CSF, IL-2, and IL-6 for 72 h) in the presence of 8803, 27907, or their combination. **F** Cytotoxicity following withdrawal of 8803: bEnd.3 cells were pre-treated (pret. 8803) with 10 µg/mL 8803 for 18 h, washed, and co-cultured with pre-activated splenocytes in the presence of medium alone, 27907, or an IgG isotype control. **C**-**F** Relative O.D., relative luminescence, or % luminescence are plotted on the y-axis. Whole cell lysate (WCL) and not-treated (NT). Statistical significance was determined by ordinary one-way ANOVA, with *P < 0.05, **P < 0.01, ***P < 0.001, ****P < 0.0001

Given the increased intratumor vasculature damage observed in vivo following combination treatment with 27907 and 8803 compared to either agent alone (Fig. 6), we investigated the potential mechanisms underlying this enhanced vascular targeting. Type I and II IFNs, which are potently induced by STING activation [2, 3], are known to upregulate immune checkpoints such as PD-L1 and PD-L2 on endothelial cells [15]. We therefore hypothesized that the STING agonist 8803 may modulate PD-L1 and/or PD-L2 expression on tumor-associated endothelial cells, thereby sensitizing them to FcγR-mediated effector functions, such as ADCC and ADCP elicited by PD-L1/PD-L2-targeting antibodies. This mechanistic interaction could contribute to the observed vascular remodeling and enhanced immune infiltration in the combination treatment group. To test this, we used bEnd.3 mouse endothelial cells, a line derived from the brain endothelium of a murine endothelioma model [36]. We first assessed whether 8803 activates the STING pathway in these cells. bEnd.3 cells were treated with 10 µg/mL of 8803 or reference STING agonists for 1 h, followed by Western blot analysis. As shown in Fig. 7A, 8803 induced phosphorylation of STING, comparable to that elicited by the known STING agonist ADU-S100, confirming robust pathway activation in endothelial cells. Next, we assessed whether STING activation by 8803 affects immune checkpoint ligand expression. Under basal conditions, both PD-L1 and PD-L2 were expressed at low levels on bEnd.3 cells. Treatment with 8803 for 18 h led to a significant, dose-dependent increase in surface PD-L1 (Fig. 7B), but not PD-L2 (Supplementary Fig. 8A), as measured by FACS. This suggests that 8803 primes endothelial cells for enhanced responsiveness to PD-L1-targeted therapies such as 27907. To explore whether IFN signaling contributes to 8803-mediated PD-L1 induction, we treated bEnd.3 cells with recombinant IFN-α, IFN-β, or IFN-γ. Each cytokine significantly increased PD-L1, but not PD-L2, expression, comparable to the effects of 8803 (Fig. 7B; Supplementary Fig. 8A). Furthermore, 8803 treatment induced IFN-α, IFN-β, and CXCL10 release in bEnd.3 cells, as assessed via supernatant analysis using cytokine reporter cell lines (Fig. 7C, D), supporting a role for autocrine or paracrine IFN signaling in PD-L1 upregulation. These findings demonstrate that STING activation by 8803 not only initiates innate immune signaling in endothelial cells but also enhances PD-L1 expression, potentially increasing their sensitivity to ADCC and ADCP. This may account for the pronounced vascular disruption and endothelial apoptosis observed in vivo with the combination therapy.

As expected, 8803 showed no important direct cytotoxicity toward bEnd.3 or splenocyte cells under the tested conditions (Supplementary Fig. 8B-D). Importantly, the splenocyte viability assays were performed using the same cytokine-preactivated splenocyte preparations used in the corresponding co-culture experiments. To evaluate whether PD-L1 upregulation sensitizes endothelial cells to immune-mediated killing, we co-cultured bEnd.3 cells with monocyte-enriched splenocytes. Combination treatment with 8803 and 27907 significantly enhanced cytotoxicity compared to either agent alone (Fig. 7E). To evaluate whether 8803-mediated PD-L1 upregulation enhances the susceptibility of endothelial cells to ADCC, we treated bEnd.3 murine endothelial cells with the STING agonist 8803 for 18 h. Following compound removal via washing, the cells were co-cultured with pre-activated murine splenocytes in the presence of either 27907 or an isotype IgG control. Cells pretreated with 8803 and subsequently exposed to 27907 exhibited significantly increased splenocyte-mediated cytotoxicity compared to cells treated with isotype control, or to groups that did not receive 8803 pre-treatment (with or without 27907; Fig. 7F and Supplementary Fig. 9). These results indicate that PD-L1 upregulation by STING activation functionally sensitizes endothelial cells to antibody-mediated killing.

### 8803 upregulates PD-L1/PD-L2 on human endothelial cells, enhancing susceptibility to 27907-mediated killing

**Fig. 8:**
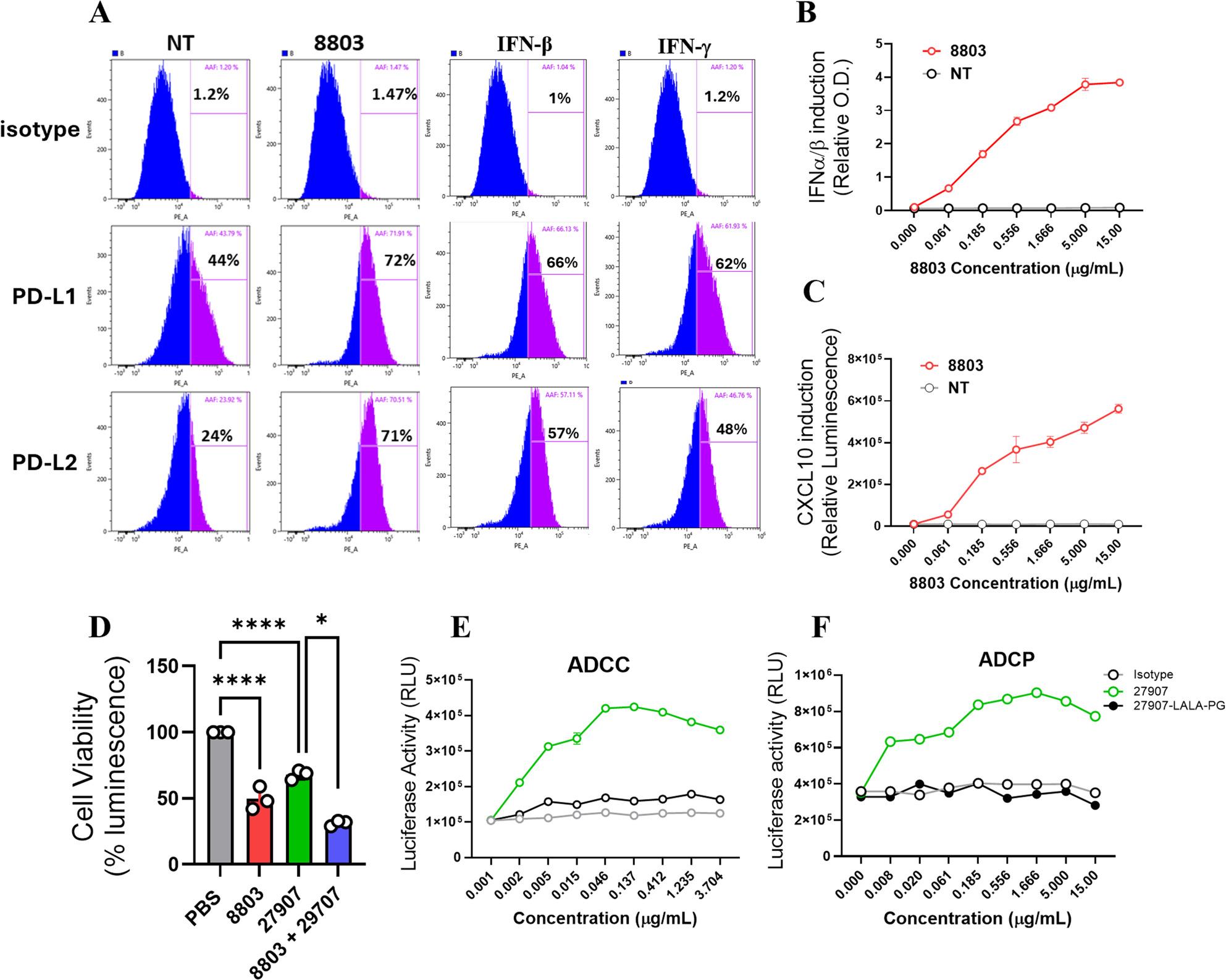
8803 upregulates PD-L1 and PD-L2 expression in human endothelial cells and enhances immune-mediated cytotoxicity. **A** FACS analysis of PD-L1 and PD-L2 surface expression on HUVECs following treatment with 8803, IFN-β, and IFN-γ. **B,** C Induction of (B) type I interferons (IFN-α/β) and (**C**) CXCL-10 production in HUVECs treated with 8803, assessed via supernatant analysis using cytokine reporter cell lines. **D** Immune-mediated cytotoxicity assay showing killing of HUVECs co-cultured with PBMCs pre-activated with GM-CSF, IL-2, and IL-6, in the presence of PBS, 8803, 27907, or their combination. **E**, **F** ADCC and ADCP assays against HUVECs in the presence of 8803 and treatment with 27907, 27907-LALA-PG, or an isotype control. **B**-**F** Relative O.D., RLU, or % luminescence are plotted on the y-axis. Statistical significance was determined by ordinary one-way ANOVA, with *P < 0.05, **P < 0.01, ***P < 0.001, ****P < 0.0001

We investigated whether 8803 modulates PD-L1 and PD-L2 expression in primary human endothelial cells. For this purpose, we used HUVECs, given their clinical relevance and established responsiveness to inflammatory stimuli [37, 38]. At baseline, HUVECs exhibited a moderate expression of PD-L1 and PD-L2. However, stimulation with 8803 for 18 h resulted in a robust, dose-dependent upregulation of PD-L1, with an even more pronounced induction of PD-L2 (Fig. 8A). This response supports a conserved mechanism of STING-mediated checkpoint ligand induction across species [39]. To explore the mechanism underlying PD-L1 upregulation, HUVECs were treated with recombinant IFNs. Both human IFN-β and IFN-γ induced high PD-L1 and PD-L2 expression. Notably, 8803 induced even higher levels of PD-L1 and PD-L2 expression (Fig. 8A), suggesting that both IFN-β and IFN-γ work in tandem to mediate STING-driven checkpoint ligand induction. Furthermore, supernatant analysis of 8803-treated HUVECs revealed increased secretion of IFN-α, IFN-β, and CXCL-10, consistent with STING-driven inflammatory activation (Fig. 8B, C). Consistently, treatment with 8803 did not impact HUVECs or PBMCs viability, as confirmed by CellTiter-Glo 2.0 assay (Supplementary Fig. 10A, B), indicating that PD-L1 and PD-L2 induction occurred independently of cytotoxic stress. Importantly, the PBMC viability assays were performed using the same cytokine-preactivated PBMC preparations used in the corresponding co-culture experiments.

To assess whether 8803-induced PD-L1 and PD-L2 expression sensitizes HUVECs to immune-mediated killing, we established a co-culture system with PBMCs from healthy donors; combination treatment with 8803 and 27907 enhanced endothelial cell killing compared to either agent alone or control (Fig. 8D). To confirm the role of Fc-mediated effector functions, we compared the activity of 27907 [23] with an Fc-silent variant, 27907-LALA-PG, in ADCC and ADCP assays. As shown in Fig. 8E, F, only 27907 antibody, when combined with 8803, significantly induced both ADCC and ADCP against HUVECs, highlighting the contribution of FcγR-mediated effector activity to the observed cytotoxicity.

## Discussion

ICB therapies targeting inhibitory pathways such as CTLA-4 and PD-1/PD-L1 have revolutionized cancer treatment, yielding durable and, in some cases, curative responses in a subset of patients, particularly those with immune-inflamed cancers such as melanoma. However, objective response rates typically range from 15% to 60% in approved indications, depending on tumor type and patient characteristics [40, 41]. In this study, we show that combining the STING agonist 8803 with the cytotoxic dual specific PD-L1/PD-L2 antibody 27907 is associated with enhanced antitumor activity in both the B16-PD-L1 melanoma and the ICB-refractory TS/A mammary carcinoma models. This combination was associated with reduced tumor growth, prolonged survival, and increased tumor necrosis. These effects are accompanied by changes in the TME, including increased immune cell infiltration and modulation of myeloid populations, consistent with a remodeling of the immunosuppressive microenvironment toward a more inflammatory state. These findings provide preliminary support for the potential utility of this approach in tumors characterized by limited immune infiltration or resistance to checkpoint blockade. Mechanistically, our data suggest that the activity of this combination may be driven, at least in part, by STING-mediated upregulation of PD-L1 and PD-L2 on tumor-associated endothelial cells, thereby increasing susceptibility to Fc-mediated effector functions triggered by 27907. The selective upregulation of PD-L1 and PD-L2 on endothelial cells, rather than exclusively on tumor cells or infiltrating immune populations, suggests a potential vascular component to the therapeutic activity of this combination. The observed induction of PD-L1 and PD-L2 in endothelial cells following 8803-mediated STING activation likely reflects a broader immune-regulatory axis, where type I IFN signaling downstream of STING upregulates checkpoint ligands. In murine models, STING activation within tumor endothelial cells drives the production of type I IFNs, particularly IFN-β, which initiates spontaneous and therapeutic antitumor CD8⁺ T-cell responses and potentiates the effects of ICB [42]. Additionally, the STING pathway is a central component of the T cell-inflamed TME, promoting type I IFN-driven immune priming within tumors and underpinning responsiveness to immunotherapy [43]. These observations support a mechanistic framework in which endothelial upregulation of PD-L1/PD-L2 following STING activation may sensitizes tumors to the PD-L1/PD-L2-targeting antibody, highlighting a crosstalk between innate STING-driven signaling and the modulation of adaptive immune checkpoints. This interaction may contribute to the vascular disruption and immune-mediated cytotoxicity observed in vivo. Indeed, 27907 provides dual checkpoint blockade and efficiently triggers Fc-dependent effector functions such as ADCC and ADCP, due to to the GASDIE mutations. Notably, 27907 targets PD-L1/PD-L2-expressing populations, including immunosuppressive subsets such as mregDCs and TAMs. Furthermore, it is associated with changes in tumor vasculature, including increased CD62p expression, endothelial cell apoptosis, and features consistent with thrombus formation. In this context, FcγR engagement by 27907 may contribute to immune-mediated clearance and tumor damage. To further assess the contribution of Fc-effector functions to the therapeutic activity of 27907, we compared the Fc-competent variant to 27907-LALA-PG, an Fc-silent variant in which effector function has been abolished through introduction of the LALA-PG mutations, both in vitro and in vivo [44]. While both variants retain checkpoint-blocking activity, only the Fc-competent variant demonstrated robust ADCC and ADCP activity in vitro*.* This difference in effector function translated into a meaningful difference in therapeutic outcome in vivo, where combination with 8803 produced a survival benefit with the Fc-competent variant, whereas the Fc-silenced variant showed only a modest effect under the same conditions. Collectively, these observations suggest that checkpoint blockade alone is insufficient to account for the full therapeutic activity of 27907, and that Fc-mediated effector functions contribute meaningfully to the antitumor response in this combination setting.

The use of both engineered and native expression models supports the interpretation of FcγR-mediated activity across varying PD-L1/PD-L2 expression settings. In addition, bio-layer interferometry analyses demonstrated that 27907 binds human and murine PD-L1 and PD-L2 with comparable high affinity, supporting the relevance of the murine models used in this study. Consistent with this, we confirmed binding of 27907 to murine PD-L1 and PD-L2 at the cellular level and demonstrated differential engagement of murine FcγRs by the engineered Fc variants. Comparative analysis of the B16-PD-L2 and TS/A tumor models revealed distinct patterns of PD-L1 and PD-L2 expression across immune cell populations. In TS/A tumors, both ligands were broadly expressed among myeloid cells, including MDSCs, DCs, and TAMs. In contrast, the B16-PD-L2 model showed widespread PD-L1 expression but more restricted PD-L2 expression, confined to low levels in TAMs and DCs. These differences likely reflect tumor-specific cytokine milieus, particularly differential TGF-β signaling [45–48]. Understanding these patterns provides further mechanistic insight into the differential responsiveness to dual ICB and highlights the importance of characterizing ligand expression profiles when designing combinatorial immunotherapy strategies.

Together, our data support a mechanistic interplay in which 8803 induces PD-L1 and PD-L2 expression via STING and IFN signaling, thereby potentially sensitizing cells within the TME, including the vasculature, to FcγR-mediated immune attack. While this mechanism is supported by both in vitro and in vivo observations, it is likely that multiple and complementary processes contribute to the overall therapeutic effect. Consistent with this, our findings align with previous reports demonstrating that STING activation within the TME promotes M2 macrophage repolarization and enhances CD8⁺ T-cell responses [42, 49]. Notably, the validity of the observed PD-L1/PD-L2 modulation is supported by competition experiments demonstrating that 27907 does not interfere with the binding of detection antibodies, thereby reducing the likelihood of confounding artifacts in FACS analyses.

Collectively, these findings indicate that 8803 enhances immune activation and modulates the TME, promoting both innate and adaptive antitumor responses. Combination therapy is associated with immunologic remodeling, including increased T cell infiltration and changes in TAM phenotype, although some of these observations derive from analyses with limited sample size and should be interpreted with caution pending further validation. These results support the therapeutic potential of concurrently targeting STING and PD-L1/PD-L2 to amplify antitumor immunity. More broadly, the results illustrate how the efficacy of STING-based therapies can be potentiated through rational combination strategies. Inflammatory activation alone is insufficient to overcome a highly suppressive TME; the addition of a targeted, Fc-competent antibody that blocks both PD-L1 and PD-L2 and promotes effector functions may enhance therapeutic outcomes. This also highlights a potential limitation of conventional PD-1/PD-L1 inhibitors in tumors where PD-L2 plays a dominant role or where FcγR engagement contributes to immune clearance.

The biological rationale for combining STING activation with immune checkpoint inhibitors is substantiated by preclinical and clinical evidence demonstrating therapeutic benefit in multiple contexts [50–52]. In a phase 1b study, combination of intratumorally administered STING agonist with the anti–PD-1 spartalizumab in patients with solid tumors showed higher overall response rate than observed with single-agent STING agonist [53, 54]. Consistent with these reports, the preclinical data presented here indicate that the combination of 8803 and a PD-L1/PD-L2 cytotoxic antibody is associated with enhanced antitumor activity and remodeling of the TME.

In summary, our findings support a revised immunotherapy paradigm that leverages the complementary actions of innate immune activation and targeted cytotoxicity [55]. By reshaping the TME and sensitizing stromal and endothelial components to immune attack, STING agonists such as 8803 may create a window of vulnerability that dual PD-L1/PD-L2-targeting antibodies such as 27907 can exploit. This combination approach may offer benefit in tumors resistant to standard ICB, with ongoing studies expected to further define its mechanisms and translational potential.

## Supplementary Information

Supplementary Material 1.

Supplementary Material 2.

Supplementary Material 3.

## Data Availability

All unique reagents and cell lines generated in this study will be made available on request with a completed materials transfer agreement and with reasonable compensation by requestor for their processing and shipping. We may require a payment if there is potential for commercial application. All data reported in this paper and any additional information required to reanalyze the data reported in this paper will be shared by the corresponding author, Federica Cavallo ( [federica.cavallo@unito.it](mailto:federica.cavallo@unito.it) ) upon request. This paper does not report original codes.

## Abbreviations

ADCC: Antibody-dependent cellular cytotoxicity
ADCP: Antibody-dependent cellular phagocytosis
CDNs: Cyclic dinucleotides
CHO: Chinese hamster ovary cells
DCs: Dendritic cell
FcγR: Fcγ receptor
FMO: Fluorescence-minus-one
HUVECs: Human umbilical vein endothelial cells
ICB: Immune checkpoint blockade
IF: Immunofluorescence
IFN: Interferon
IHC: Immunohistochemistry
i.p.: Intraperitoneal
IRFs: Interferon regulatory factors
i.t.: Intratumor
MDSCs: Myeloid-derived suppressor cells
MFI: Mean Fluorescence Intensity
mregDCs: Mature regulatory dendritic cells
NF-κB: Nuclear factor kB
NK: Natural killer
PBMCs: Human peripheral blood mononuclear cells
PD-1/PD-2: Programmed cell death protein-1(-2)
PD-L1/PD-L2: Programmed cell death protein-1(2) ligand
s.c.: Subcutaneously
RLU: Relative luminescence units
STAT3: Signal transducer and activator of transcription
STING: Stimulator of interferon genes
TAMs: Tumor-associated macrophages
THP1-KO: THP1-Dual™ STING knockout cells
TME: Tumor microenvironment
Tregs: Regulatory T cells
