# Supplementary material for: Tumor microenvironment remodeling by STING agonism sensitizes endothelial cells to cytotoxic anti-PD-L1/L2 antibody
Source: J Exp Clin Cancer Res. 2026 Apr 14;45:125. doi: 10.1186/s13046-026-03711-9 (PMC13196009; doi:10.1186/s13046-026-03711-9)
Supplement: Supplementary file 1 — Supplementary Material 1. [file 13046_2026_3711_MOESM1_ESM.docx]

**Supplementary Figures from**

**Tumor Microenvironment Remodeling by STING Agonism Sensitizes Endothelial Cells to Cytotoxic Anti-PD-L1/L2 Antibody**

Ahmad Salameh^1^, Elisabetta Bolli^2^, Manuela Iezzi^3, 4^, Christine Gagliardi^1^, Laura Conti^2^, Chiara Cossu^2^, Paul Blezinger^1^, Alessia Lamolinara^3^, Andrew Lewis^1^, Michael A. Curran^5^, Federica Cavallo^2^*, and Federica Pericle^1^.

*^1^ImmunoGenesis, Inc., Houston, TX, USA*

*^2^Laboratory of OncoImmunology, Molecular Biotechnology Center “Guido Tarone”, Department of Molecular Biotechnology and Health Sciences, University of Turin, Turin, Italy*

*^3^Laboratory of Experimental Pathology and Precision Medicine, Center for Advanced Studies and Technology (CAST), Department of Neurosciences, Imaging and Clinical Sciences, "G. d'Annunzio University of Chieti-Pescara, Chieti, Italy*

*^4^Eusoma Breast Centre, Department of Pathology, "G. Bernabeo" Hospital Ortona, ASL2 Abruzzo, Ortona, Italy*

*^5^Department of Immunology, The University of Texas MD Anderson Cancer Center, Houston, Texas, USA*

*Corresponding author: Federica Cavallo, Molecular Biotechnology Center “Guido Tarone”, Piazza Nizza 44b, 10126, Turin, Italy. E-mail: federica.cavallo@unito.it


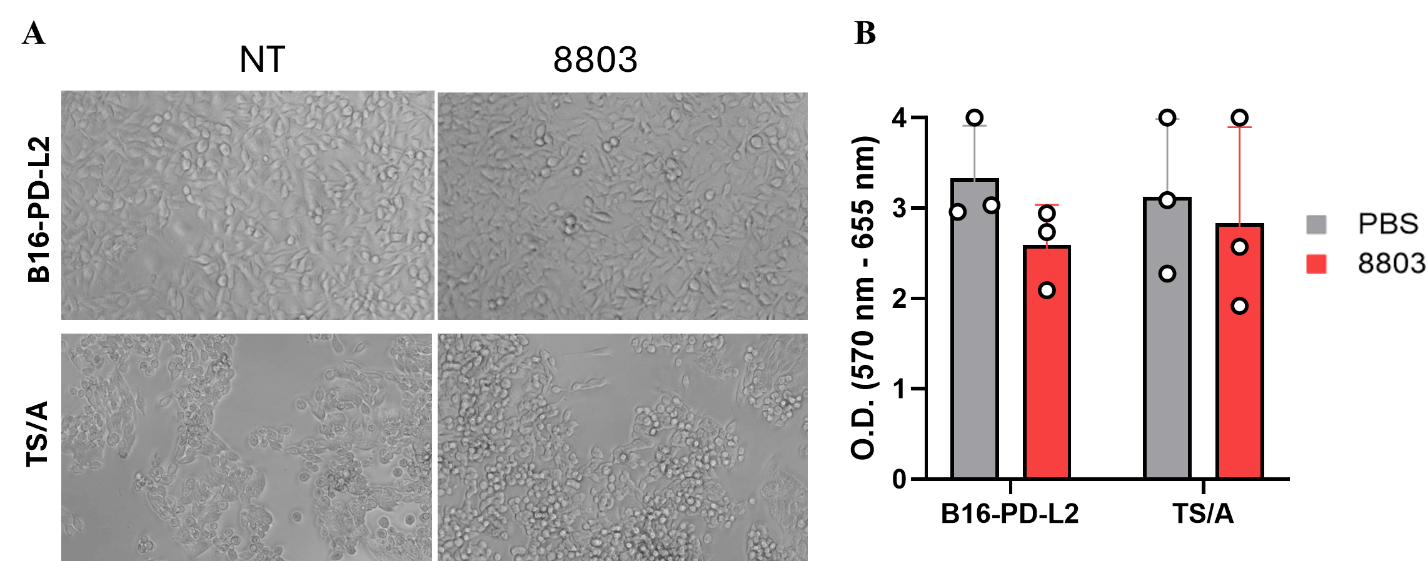


**Supplementary Figure 1. 8803 does not affect tumor cell viability *in vitro.*** (**A**) Representative images of B16-PD-L2 and TS/A tumor cells after 48 hours incubation with the STING agonist 8803 (10 µg/ml). Cell viability at 48 hours of incubation with 8803 (10 µg/ml) assessed by MTT assay. (**B**) Optical densities (O.D.) of dissolved formazan crystals are used as a redout of cell number.


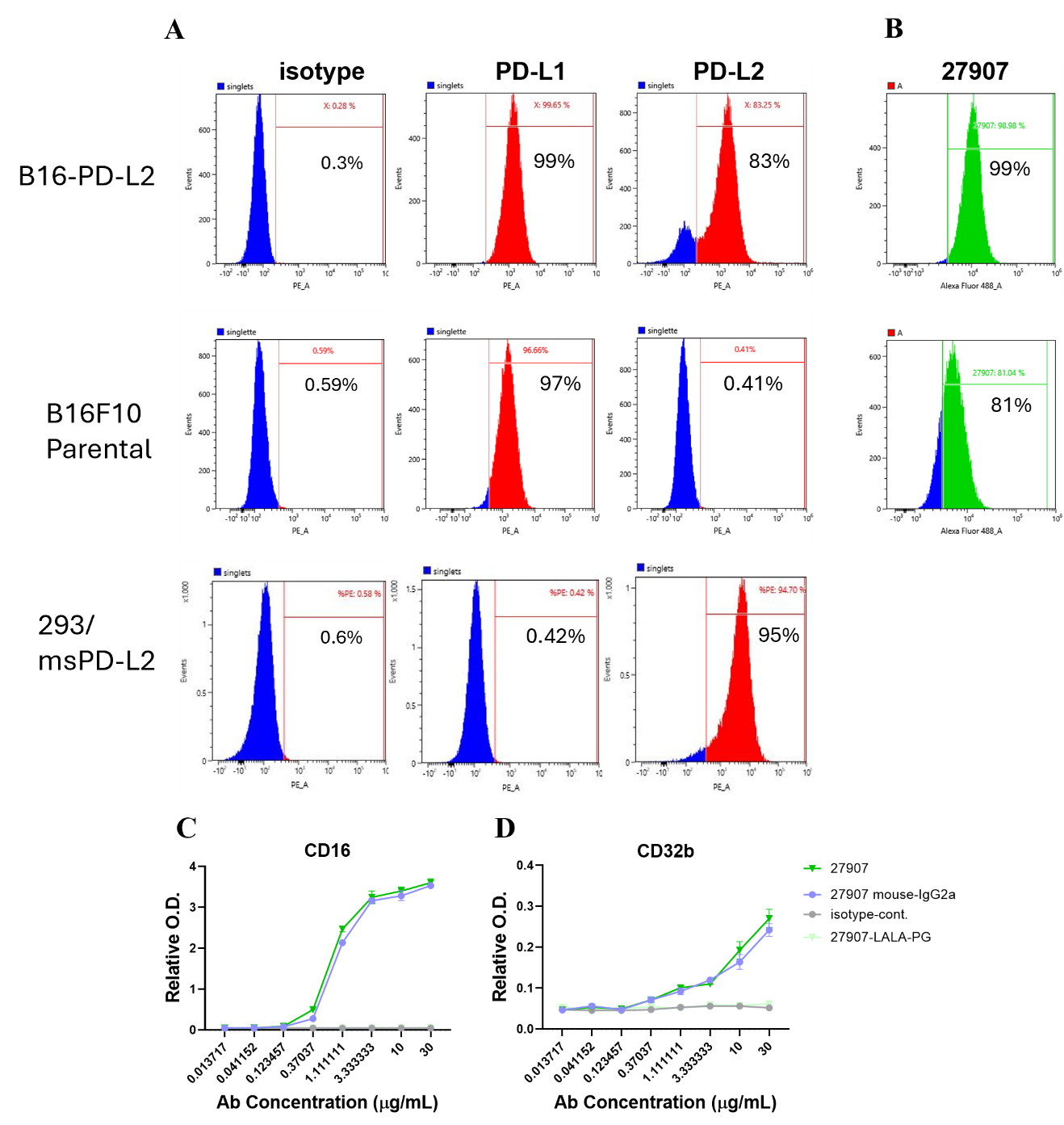


**Supplementary Figure 2. FACS analysis of PD-L1 and PD-L2 expression on B16-PD-L2, B16F10 parental, and 293/msPD-L2 cell lines. (A)** Surface expression of PD-L1 and PD-L2 on B16-PD-L2, B16F10 parental, and 293 cells expressing mouse PD-L2 (293/mPD-L2), assessed by FACS using commercially available antibodies against PD-L1 (clone 10F.9G2) and PD-L2 (clone TY25), with appropriate isotype controls. **(B)** Binding of 27907 to B16-PD-L2 and B16F10 parental cells. **(C, D)**FcγR binding characterization of 27907 and isotype variants by ELISA. 27907 antibodies were serially diluted (30 – 0.12 µg/mL) and immobilized on ELISA (pre-coated plates with 50 ng/well). Biotinylated FcγRs (CD16 or CD32b) were added, followed by ExtrAvidin-HRP detection. Binding was quantified by HRP substrate absorbance.


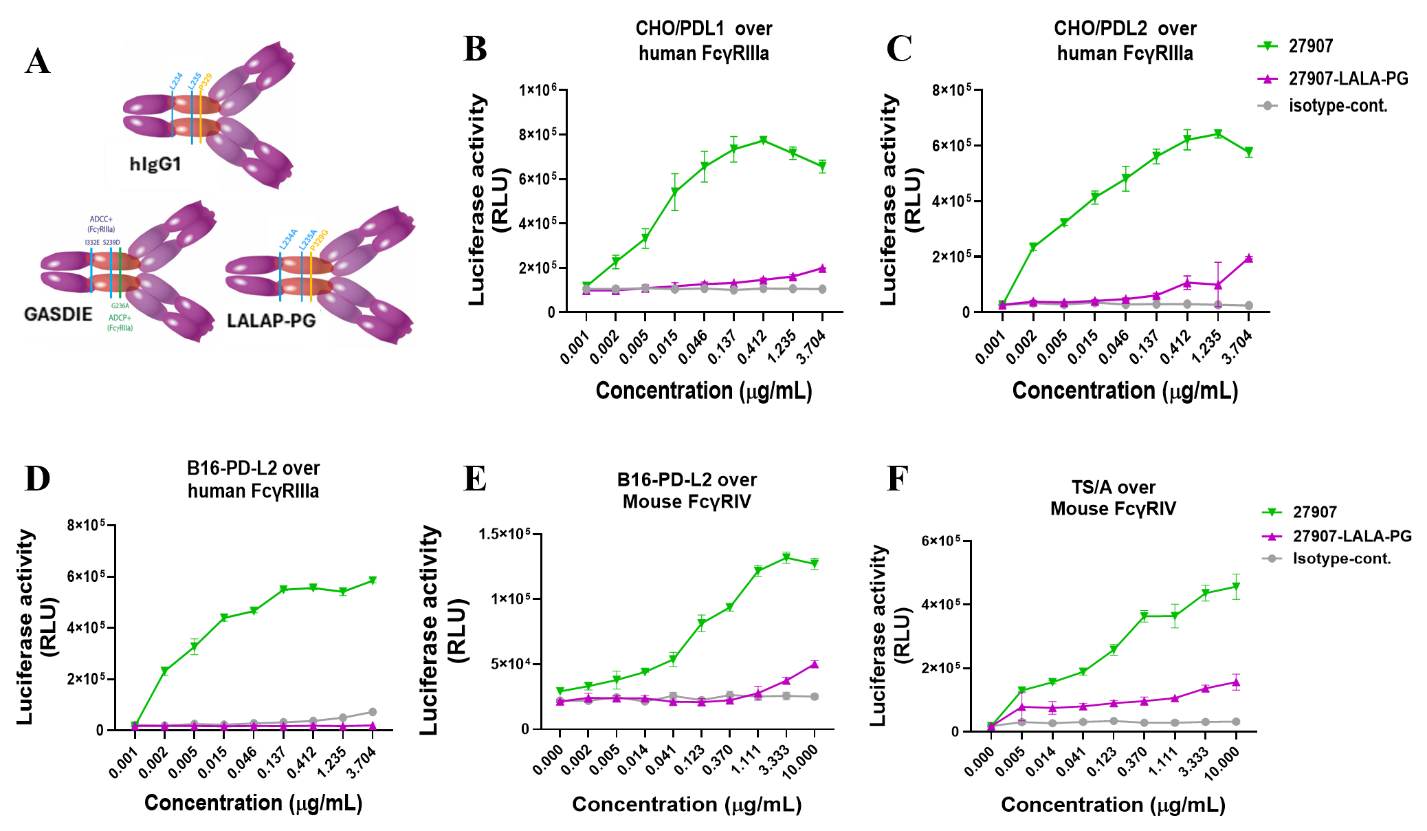


**Supplementary Figure 3. Assessment of FcγRIIIa engagement by Fc-engineered antibody variants (GASDIE, LALA-PG) *in vitro*.** (**A**) Schematic representation of the dual-specific human IgG1 antibody and its Fc-engineered variants. The GASDIE (G236A/S239D/I332E) and LALA-PG (L234A/L235A/P329G) mutations were introduced into the Fc region to modulate Fc-mediated effector functions. GASDIE enhances FcγR engagement and effector activity, whereas LALA-PG reduces Fc-mediated functions such as ADCC and ADCP. (**B–F**) 27907-GASDIE and -LALA-PG were evaluated for their ability to engage human FcγRIIIa and mouse FcγRIV and induce ADCC, using a reporter bioassay. Target cells were CHO/PD-L1 (**B**), CHO/PD-L2 (**C**), B16-PD-L2 (**D,** **E**) and TS/A (**F**) cells. Effector cells consisted of Jurkat T cells engineered to express an NFAT-RE luciferase reporter together with human (**B-D**) or mouse FcγRIV (**E, F**). Following co-culture, luciferase activity was quantified as a readout of FcγR engagement. Differences in reporter signal among antibody variants reflect their relative capacity to engage FcγRs under these conditions. A higher luminescence signal expressed as relative luminesce units (RLU) indicates an efficient ADCC activation pathway. Mean ± SEM (n = 3) is displayed for each concentration of each antibody.

**Supplementary Figure 4. Characterization of PD-L1 and PD-L2 expression in B16-PD-L2 tumors, myeloid gating strategy, and binding competition between the therapeutic antibody 27907 and staining antibodies.** (**A**) Assessment of binding competition between the therapeutic anti–PD-L1/PD-L2 antibody 27907 and staining antibodies. B16-PD-L2 cells were pre-incubated with a saturating concentration of 27907 (30 µg/mL) prior to staining with anti–PD-L1 (clone 10F.9G2) and anti–PD-L2 (clone TY25). (**B**) Representative FACS density plots showing the myeloid gating strategy. Live, singlet CD45⁺ cells were gated and subdivided into monocytic (m)MDSCs (CD11b⁺Ly6C⁺Ly6G⁻), granulocytic (g)MDSCs (CD11b⁺Ly6G⁺Ly6C^low^), TAMs (CD11b⁺Ly6G⁻Ly6C⁻CD206⁺) and dendritic cells (DCs, CD11c^+^). Ly6G⁻Ly6C⁻F4/80⁺ double-negative (DN) cells. (**C–D**) FACS analysis showing PD-L1 and PD-L2 expression in single-cell suspensions from B16-PD-L2 tumors, gated on CD45⁺ immune infiltrates and CD45⁻ tumor/stromal cells.


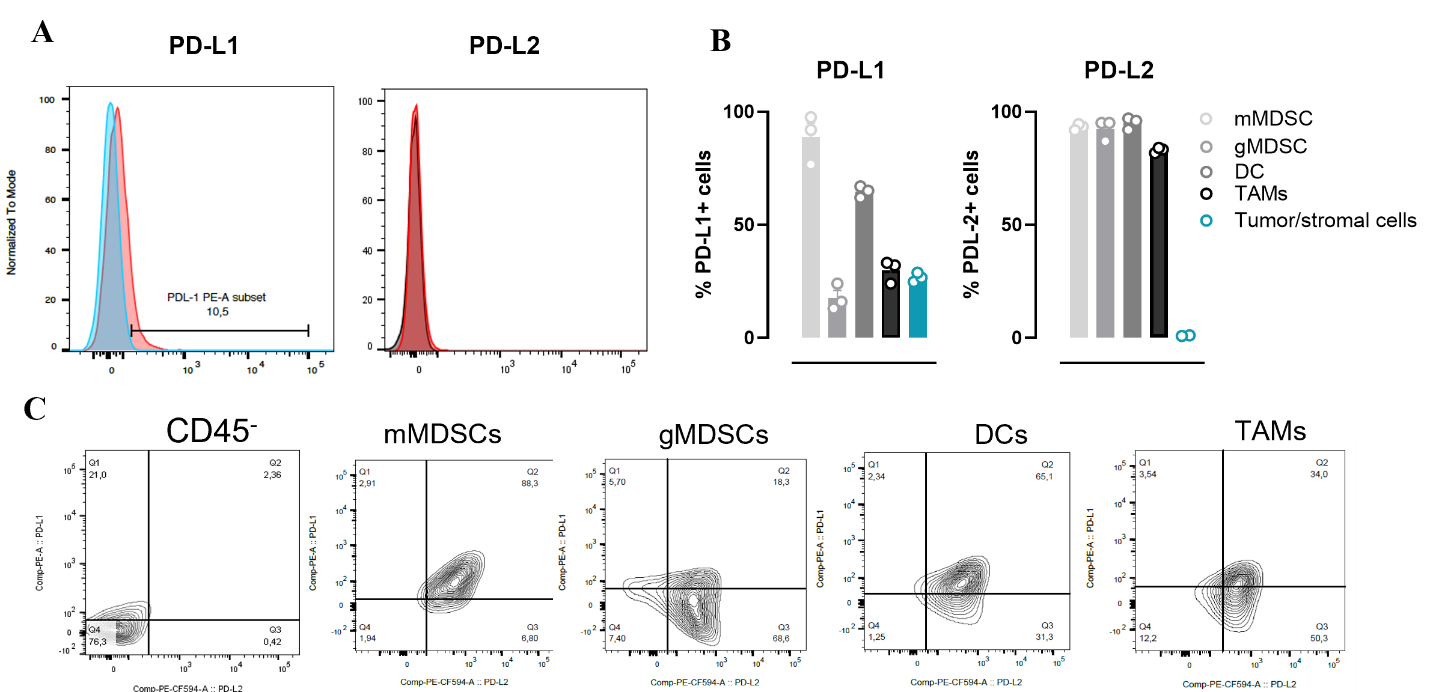


**Supplementary Figure 5. Analysis of PD-L1 and PD-L2 expression in TS/A cell line and within the TME.** **(A)** FACS analysis of PD-L1 and PD-L2 surface expression on TS/A cells cultured in vitro. **(B)** Quantification of PD-L1 and PD-L2 expression on immune cell subsets infiltrating TS/A tumors, shown as the percentage of positive cells within each subset. **(C)** Representative density plots displaying PD-L1 and PD-L2 expression on tumor and stromal cells, mMDSCs, gMDSCs, DCs, and TAMs. FACS analysis was performed using the gating strategy described in Supplementary Figure 4B.

**Supplementary Figure 6. Combination of 27907 and 8803 TME effects in the IBC-resistant TS/A model.** BALB/c mice were injected orthotopically with 1 x 10^5^ TS/A cells into the fourth left mammary gland. When the tumor reached 75-100 mm^3^, mice were randomized in the treatment groups and were injected intratumorally 2 times at 3 - 4 days intervals either with 10 µL of PBS or 10 µg of 8803 and treated i.p. with either 100 µL of PBS alone (untreated) or containing 200 µg of 27907, twice a week for three weeks. Mice were sacrificed 3 - 4 days after the final treatment and tumors were excised and processed for analysis of the TME through histology and immunohistochemical/fluorescence analysis. **(A**, **C**, **E)** Representative IHC images of tumors stained with anti-CD3 to identify T cells, anti-Foxp3 for regulatory T cells, and anti-F4/80 for macrophages. **(B**, **D**, **F)** Dot plots displaying the percentage of positive cells in tumors under different treatments. **(G**, **I)** Representative immunofluorescence images of tumors stained with anti-F4/80 to identify macrophages, anti-CD206 as a marker for M2 polarization, and DAPI for visualizing nuclei. **(H**, **J).** Histograms illustrating the percentage of positive CD206 macrophages and the intensity of CD206 fluorescence in tumors with various treatments. Statistical significance was determined by ordinary one-way ANOVA, with *P < 0.05, **P < 0.01, ***P < 0.001, ****P < 0.0001.

**Supplementary Figure 7. PD-L1 and PD-L2 expression in the TME and Tumor Draining Lymph Nodes (TDLNs) of B16-PD-L2 tumors.** FACS analysis of PD-L1 and PD-L2 surface expression on TME and TDLN. Percentage of PD-L1^hi^ (**A**), PD-L1 (**B**) and PD-L2 (**C**) expression in mMDSCs, gMDSCs, TAMs (macrophages in TDLNs), and DCs. Data are presented as mean ± SEM. Statistical significance was assessed using ordinary one-way ANOVA with *P < 0.05, **P < 0.01, ***P < 0.001, ****P < 0.0001.

**Supplementary Figure 8. 8803 does not alter PD-L2 expression or viability in mouse endothelial cells.** (**A**) FACS analysis of surface PD-L2 expression on bEnd.3 cells following 8803 treatment compared with untreated controls or treatment with IFNα, IFNβ, and IFNγ. (**B**) Cell viability assessed by 7-AAD staining showing no significant increase in cell death after stimulation with 8803. For functional assessment, bEnd.3 cells (**C**) and mouse splenocytes (**D**) were cultured with or without 10 µg/mL 8803 for 48 hours and 50 ng/ml of IFNα, IFNβ, or IFNγ for 18 hours. Cell viability was measured using the CellTiter-Glo Luminescent Cell Viability Assay, and luminescence is presented as percent luminescence.

**
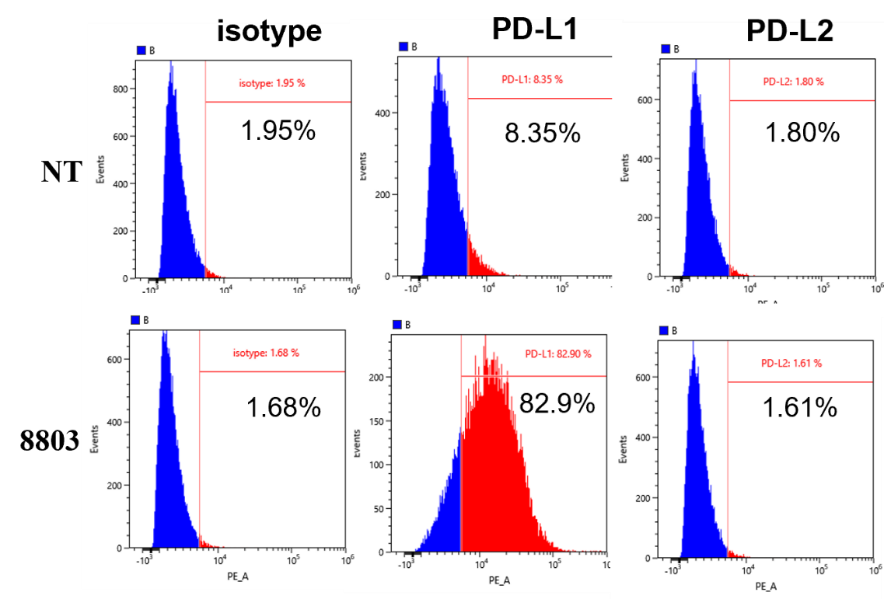
**

**Supplementary Figure 9. Flow cytometric analysis of PD-L1 and PD-L2 expression on bEnd.3 cells following 8803 treatment.** bEnd.3 cells were treated with 8803 for 18 h, after which the treatment was removed, and cells were cultured for additional 24 hours. Surface PD-L1 and PD-L2 expression was then analyzed by FACS.

**Supplementary Figure 10. Evaluation of 8803 effect on cell viability of HUVECs and human PBMCs.** Luminescence was measured after 48 hours in the presence or absence of 10 µg/mL of 8803. HUVECs (**A**) PBMCs from healthy donor (**B**). Percentage of luminescence (using the CellTiter-Glo Luminescent Cell Viability Assay) shown on the y-axis.
