# Supplementary material for: Tumor microenvironment remodeling by STING agonism sensitizes endothelial cells to cytotoxic anti-PD-L1/L2 antibody
Source: J Exp Clin Cancer Res. 2026 Apr 14;45:125. doi: 10.1186/s13046-026-03711-9 (PMC13196009; doi:10.1186/s13046-026-03711-9)
Supplement: Supplementary file 2 — Supplementary Material 2. [file 13046_2026_3711_MOESM2_ESM.docx]

**Supplementary Methods, Key Resources**

**Tumor Microenvironment Remodeling by STING Agonism Sensitizes Endothelial Cells to Cytotoxic Anti-PD-L1/L2 Antibody**

Ahmad Salameh^1^, Elisabetta Bolli^2^, Manuela Iezzi^3, 4^, Christine Gagliardi^1^, Laura Conti^2^, Chiara Cossu^2^, Paul Blezinger^1^, Alessia Lamolinara^3^, Andrew Lewis^1^, Michael A. Curran^5^, Federica Cavallo^2^*, and Federica Pericle^1^.

*^1^ImmunoGenesis, Inc., Houston, TX, USA*

*^2^Laboratory of OncoImmunology, Molecular Biotechnology Center “Guido Tarone”, Department of Molecular Biotechnology and Health Sciences, University of Turin, Turin, Italy*

*^3^Laboratory of Experimental Pathology and Precision Medicine, Center for Advanced Studies and Technology (CAST), Department of Neurosciences, Imaging and Clinical Sciences, "G. d'Annunzio University of Chieti-Pescara, Chieti, Italy*

*^4^Eusoma Breast Centre, Department of Pathology, "G. Bernabeo" Hospital Ortona, ASL2 Abruzzo, Ortona, Italy*

*^5^Department of Immunology, The University of Texas MD Anderson Cancer Center, Houston, Texas, USA*

*Corresponding author: Federica Cavallo, Molecular Biotechnology Center “Guido Tarone”, Piazza Nizza 44b, 10126, Turin, Italy. E-mail: federica.cavallo@unito.it

***Cell Viability Assays***

Cell viability was assessed using the MTT colorimetric assay or the CellTiter-Glo® 2.0 luminescent assay (Promega; Cat#G9241), depending on the cell type. For all assays, cells were seeded in 96-well plates and cultured overnight at 37 °C in a humidified incubator with 5% CO₂ prior to treatment with 8803 (10 µg/mL) for 48 hours.

For tumor cell lines (B16-PD-L2 and TS/A) and endothelial cells (bEnd.3 and HUVECs), cells were seeded at 1 × 10⁴ cells/well. For mouse splenocytes and human peripheral blood mononuclear cells (PBMCs), cells were seeded at 1 × 10⁵ cells/well; where applicable, the same cell preparations used in corresponding co-culture experiments were employed, including cytokine pre-activation with granulocyte-macrophage colony-stimulating factor (GM-CSF), interleukin (IL)-2, and IL-6.

For the MTT assay, 3-(4,5-dimethylthiazol-2-yl)-2,5-diphenyltetrazolium bromide (MTT; 0.5 mg/mL; Merck) was added to each well and incubated for 4 hours at 37 °C. The supernatant was carefully removed, and 150 µL of dimethyl sulfoxide (DMSO; Merck) was added to dissolve the formazan crystals. Absorbance was measured at 570 nm with background correction at 650 nm using a Bio-Rad 680XR microplate reader. For the CellTiter-Glo® 2.0 assay, an equal volume of reagent was added to each well, plates were shaken briefly to induce cell lysis, and luminescence, proportional to intracellular ATP levels, was measured after a 10-minute incubation at room temperature using a BioTek Cytation™ 5 Cell Imaging Multi-Mode Reader. For both assays, cell viability was calculated relative to untreated control wells.

In parallel, phase-contrast microscopy was performed on B16-PD-L2 and TS/A cells seeded in 6-well plates and treated with 8803 (10 µg/mL) for 48 hours. Images were captured at x10 magnification using a Leica DMi1 inverted microscope equipped with a DC120 digital camera.

***Cell Lines***

B16-PD-L2 cells were generated by transfecting B16F10 cells with mouse PD-L2, followed by fluorescence-activated cell sorting. B16F10 parental cell line was purchased from ATCC (Cat#CRL-6475). Human embryonic kidney 293 cells (HEK 293) were transduced with lentiviral particles encoding mouse PD-L2 (Pdcd1lg2; Cat# MR222499L3V, OriGene Technologies) to generate 293/msPD-L2 cells. All cell lines were cultured at 37°C in a humidified atmosphere containing 5% CO₂ and tested negative for mycoplasma contamination using the MycoAlert™ Mycoplasma Detection Kit (Lonza, Cat#LT07-318).

***ELISA***

Fcγ receptor (FcγR) binding of 27907 antibody isotype variants was evaluated using an ELISA-based assay. Antibodies were diluted in 0.1 M bicarbonate buffer (pH 9.0) using a 1:3 serial dilution starting at 30 µg/mL to generate final concentrations of 30, 10, 3.3, 1.1, 0.37, and 0.12 µg/mL. A volume of 100 µL per well was added to ELISA plates and incubated either overnight at 4 °C or for 2 hours at room temperature to allow adsorption of antibodies to the plate surface. Plates were washed three times with PBS containing 0.05% Tween-20 (PBST) and blocked with SuperBlock™ Blocking Buffer (Thermo Fisher Scientific; Cat# 37535) for 1 hour at room temperature. After removal of the blocking buffer, biotinylated mouse FcγRs diluted in 5% BSA were added at 50 ng/well and incubated for 2 hours at room temperature. The following recombinant proteins were used: Biotinylated Mouse FcγRIII/CD16 Protein (His, Avitag™; Cat#FC6-M82E0) and Biotinylated Mouse FcγRIIb/CD32b Protein (Avitag™, His Tag; Cat#CDB-M82E8), both from ACROBiosystems. After incubation, plates were washed three times with PBST and incubated with ExtrAvidin–HRP (1 µg/mL; 1:1000 dilution) for 60 min at room temperature with constant shaking. Plates were then washed five additional times with PBST, and HRP substrate solution was added. Absorbance was measured using a microplate reader according to the substrate manufacturer’s instructions.

***ADCC Bioassays***

ADCC was assessed using commercially available reporter bioassay kits from Promega: human FcγRIIIa ADCC reporter bioassay (Cat#G7015) and mouse FcγRIV ADCC reporter bioassay (Cat#M1201). All assays were performed according to the manufacturer’s instructions. Effectors consisted of Jurkat reporter cells engineered to express human FcγRIIIa or mouse FcγRIV for ADCC enabling luciferase-based quantification of Fc receptor engagement. As target cells, CHO/PD-L1, CHO/PD-L2, B16-PD-L2, and TS/A cell lines were used. In these assays, effector activity was evaluated in the presence of test antibody 27907, 27907-LALA-PG and isotype control antibody.

***FACS analysis***

Representative gating strategies are shown in Supplementary Figure 4B. Briefly, live singlet CD45⁺ cells were first selected and then subdivided using lineage markers. Monocytic (m)MDSCs were defined as CD11b⁺Ly6C⁺Ly6G⁻, granulocytic (g)MDSCs as CD11b⁺Ly6G⁺Ly6C^low^, and TAMs as CD11b⁺Ly6G⁻Ly6C⁻F4/80⁺. Quantification of PD-L1, PD-L2 and CD206 expression was performed calculating i) the percentage of positive cells above the fluorescence-minus-one (FMO) or isotype control threshold in the corresponding parent populations, and ii) the Mean Fluorescence Intensity (MFI) for the positive populations, to assess expression level per cell. Data were acquired using a Sony 3800 or a BD FACSVerse flow cytometers and analyzed using FlowJo v10. Compensation and instrument settings were standardized across all samples to ensure comparability of MFI measurements. For *in vitro* staining of PD-L1 and PD-L2, B16-PD-L2 or TS/A cells were detached from culture flasks, washed with PBS containing 0.5% BSA, and 0.5 × 10⁶ cells were stained with anti-mouse PD-L1-PE, PD-L2-PE or control isotype antibodies. To assess the binding of 27907 to tumor cells, 0.5 × 10⁶ cells were incubated with 2 μg of 27907 for 30 minutes on ice, followed by washing and secondary staining with an Alexa Fluor488-conjugated anti-human IgG antibody. Samples were acquired using a BD FACSVerse flow cytometer or NovoCyte flow cytometer and analyzed with FlowJo software**.**

***IHC analysis***

TS/A tumors were fixed in 10% neutral buffered formalin and embedded in paraffin; slides were sectioned at 5-µm thickness and stained with Hematoxylin (Bio-Optica) and Eosin (Bio-Optica) for histological examination***.*** Histological evaluation was performed using light microscopy to assess tumor architecture, viability, and the presence of necrotic regions. Necrosis was defined as areas of eosinophilic, anuclear tissue, often with ghost outlines of cells and surrounding inflammatory infiltrates. Digital images were captured using a (Nikon Eclipse Ci-L) and quantified. For immunohistochemical evaluation, slides were deparaffinized, serially rehydrated and, after the antigen retrieval procedure (microwave citrate buffer, pH 6.0, 10 minutes), incubated with the following primary antibodies: rabbit anti-mouse F4-80 antibody (70076, Cell Signalling), rabbit anti-mouse CD3 antibody (ab16669, Abcam), and anti-mouse Foxp3 (BioLegend, Cat#126403), according to the manufacturer’s protocols, followed by the appropriate secondary antibodies (Jackson Laboratories). Immunoreactive antigens were detected using streptavidin peroxidase (Thermo Scientific) and the DAB Chromogen System (Dako). After chromogen incubation, slides were counterstained in Hematoxylin (Bio-Optica) and images were scanned with Nanozoomer scanner from Hamamatsu. The percentage of CD3, Foxp3 and F4-80 positive cells was evaluated on whole tumor section (n = 2 - 3 samples per group) and analyzed with Qu-Path 0.3.2 software using positive cell detection tool. Statistical differences between the experimental groups were evaluated by applying unpaired Student’s t-test. Differences were statistically significant when p values were less than 0.05. The percentage of positive cells was plotted using the GraphPad Prism10 software (GraphPad).
