# Supplementary material for: Tumor microenvironment remodeling by STING agonism sensitizes endothelial cells to cytotoxic anti-PD-L1/L2 antibody
Source: J Exp Clin Cancer Res. 2026 Apr 14;45:125. doi: 10.1186/s13046-026-03711-9 (PMC13196009; doi:10.1186/s13046-026-03711-9)
Supplement: Supplementary file 3 — Supplementary Material 3. [file 13046_2026_3711_MOESM3_ESM.docx]

**Uncropped Western Blots from**

**Tumor Microenvironment Remodeling by STING Agonism Sensitizes Endothelial Cells to Cytotoxic Anti-PD-L1/L2 Antibody**

Ahmad Salameh^1^, Elisabetta Bolli^2^, Manuela Iezzi^3, 4^, Christine Gagliardi^1^, Laura Conti^2^, Chiara Cossu^2^, Paul Blezinger^1^, Alessia Lamolinara^3^, Andrew Lewis^1^, Michael A. Curran^5^, Federica Cavallo^2^*, and Federica Pericle^1^.

*^1^ImmunoGenesis, Inc., Houston, TX, USA*

*^2^Laboratory of OncoImmunology, Molecular Biotechnology Center “Guido Tarone”, Department of Molecular Biotechnology and Health Sciences, University of Turin, Turin, Italy*

*^3^Laboratory of Experimental Pathology and Precision Medicine, Center for Advanced Studies and Technology (CAST), Department of Neurosciences, Imaging and Clinical Sciences, "G. d'Annunzio University of Chieti-Pescara, Chieti, Italy*

*^4^Eusoma Breast Centre, Department of Pathology, "G. Bernabeo" Hospital Ortona, ASL2 Abruzzo, Ortona, Italy*

*^5^Department of Immunology, The University of Texas MD Anderson Cancer Center, Houston, Texas, USA*

*Corresponding author: Federica Cavallo, Molecular Biotechnology Center “Guido Tarone”, Piazza Nizza 44b, 10126, Turin, Italy. E-mail: federica.cavallo@unito.it

**Uncropped version of Western Blot depicted in Figure 1 E**

**
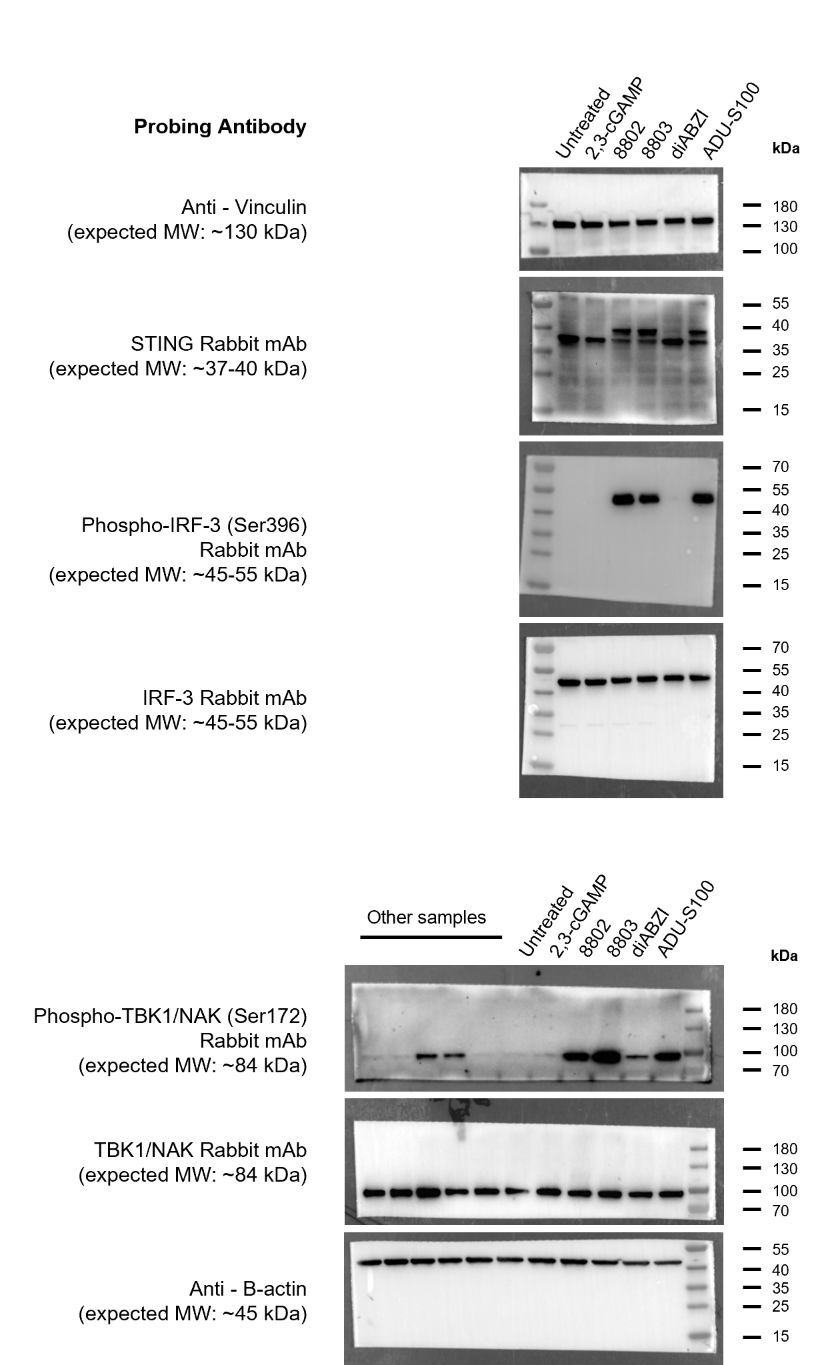
**

For the analysis of the signaling pathway, several proteins were detected on the same membrane. To include all samples and pathway components, multiple wells of the same gel were loaded with the respective samples. The membrane was sliced horizontally according to the molecular weight of the target proteins, and each part was probed with the corresponding primary antibody indicated in the figure. Loading controls (Vinculin and β-Actin) were detected on the same membrane. Chemiluminescence and colorimetric images were merged to better visualize the specific bands in relation to the ladder (Thermo Fisher Scientific, Cat# 26616). Experimental details on western blot procedures, antibodies, and imaging are reported in the Materials & Methods section.

**Uncropped version of Western Blot depicted in Figure 1 F**

**
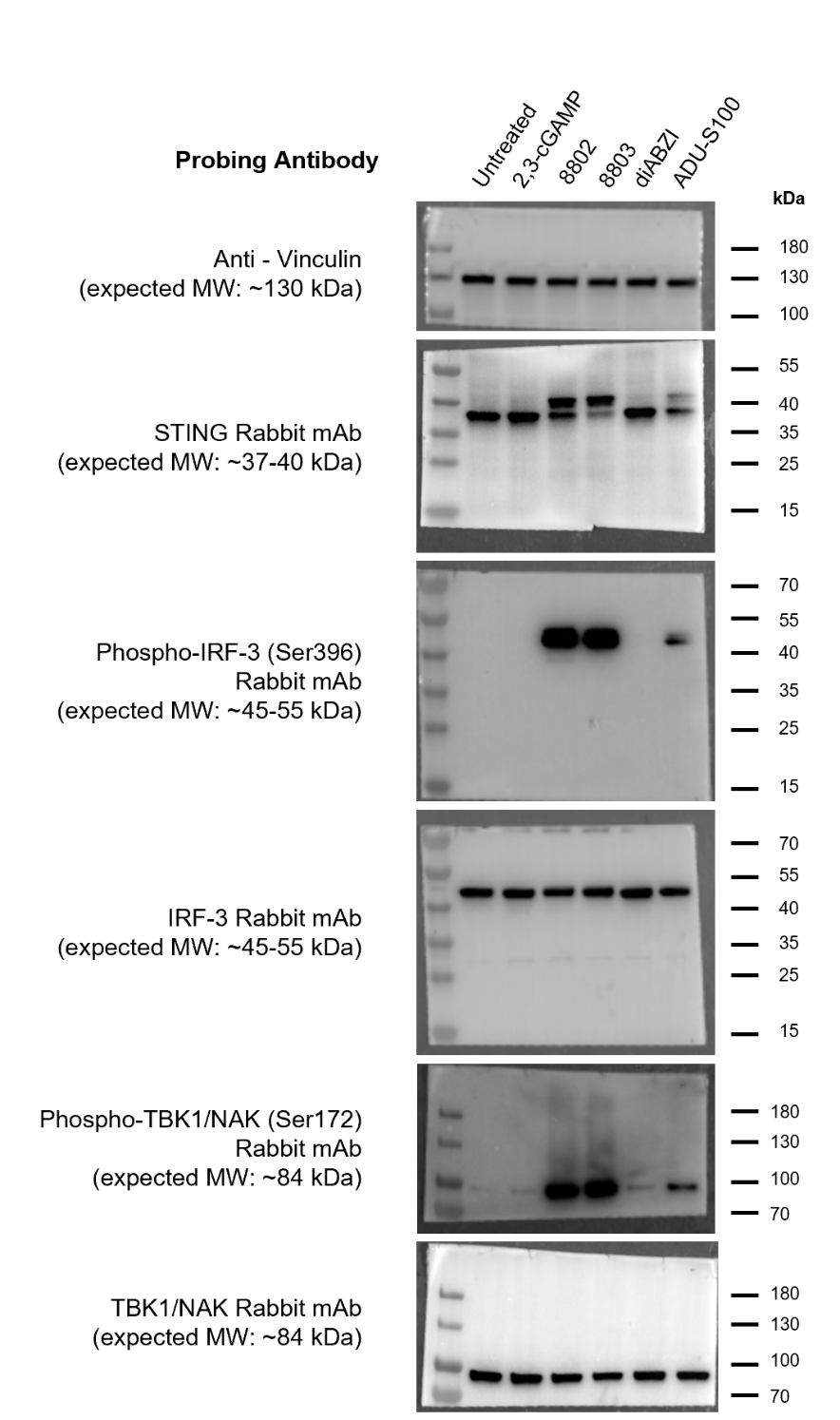
**

For the analysis of the signaling pathway, several proteins were detected on the same membrane. To include all samples and pathway components, multiple wells of the same gel were loaded with the respective samples. The membrane was sliced horizontally according to the molecular weight of the target proteins, and each part was probed with the corresponding primary antibody indicated in the figure. Loading control (Vinculin) was detected on the same membrane. Chemiluminescence and colorimetric images were merged to better visualize the specific bands in relation to the ladder (Thermo Fisher Scientific, Cat# 26616). Experimental details on western blot procedures, antibodies, and imaging are reported in the Materials & Methods section.

**Uncropped version of Western Blot depicted in Figure 7 A**

**
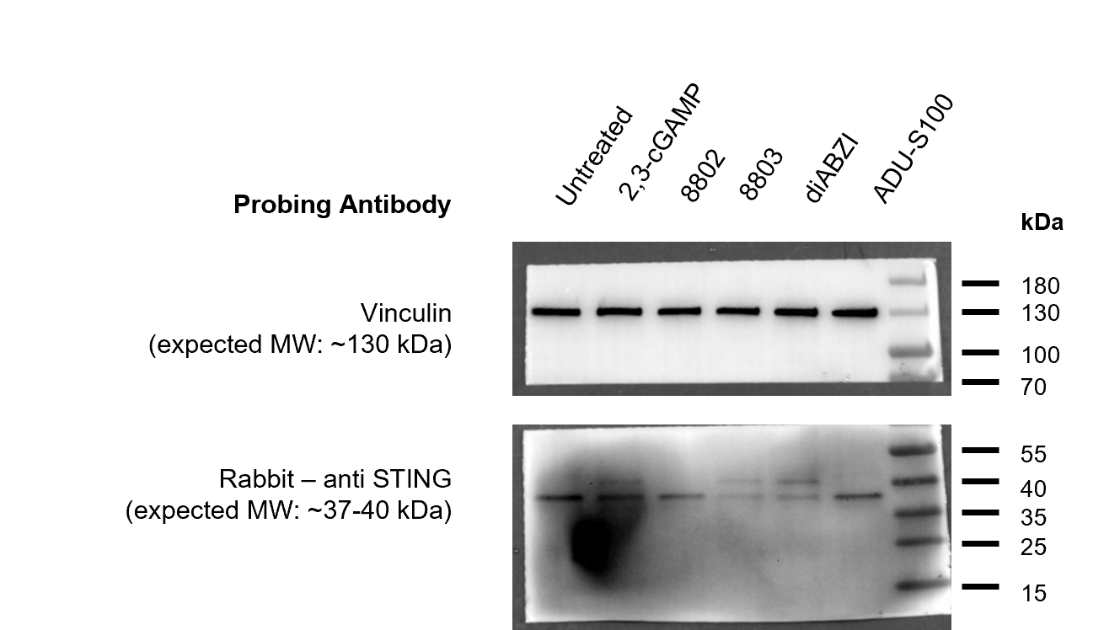
**

The membrane was sliced horizontally according to the molecular weight of the target proteins, and each part was probed with the corresponding primary antibody indicated in the figure. Loading control (Vinculin) was detected on the same membrane. Chemiluminescence and colorimetric images were merged to better visualize the specific bands in relation to the ladder (Thermo Fisher Scientific Cat# 26616). Experimental details on western blots and antibodies used are reported in the Materials & Methods section.
